# Transcriptional pause extension benefits the stand-by rather than catch-up Rho-dependent termination

**DOI:** 10.1093/nar/gkad051

**Published:** 2023-02-10

**Authors:** Eunho Song, Seungha Hwang, Palinda Ruvan Munasingha, Yeon-Soo Seo, Jin Young Kang, Changwon Kang, Sungchul Hohng

**Affiliations:** Department of Physics and Astronomy, and Institute of Applied Physics, Seoul National University, Seoul 08826, Republic of Korea; Department of Chemistry, Korea Advanced Institute of Science and Technology, Daejeon 34141, Republic of Korea; Department of Biological Sciences, Korea Advanced Institute of Science and Technology, Daejeon 34141, Republic of Korea; Department of Biological Sciences, Korea Advanced Institute of Science and Technology, Daejeon 34141, Republic of Korea; Department of Chemistry, Korea Advanced Institute of Science and Technology, Daejeon 34141, Republic of Korea; Department of Biological Sciences, Korea Advanced Institute of Science and Technology, Daejeon 34141, Republic of Korea; KAIST Stem Cell Center, Korea Advanced Institute of Science and Technology, Daejeon 34141, Republic of Korea; Department of Physics and Astronomy, and Institute of Applied Physics, Seoul National University, Seoul 08826, Republic of Korea

## Abstract

Transcriptional pause is essential for all types of termination. In this single-molecule study on bacterial Rho factor-dependent terminators, we confirm that the three Rho-dependent termination routes operate compatibly together in a single terminator, and discover that their termination efficiencies depend on the terminational pauses in unexpected ways. Evidently, the most abundant route is that Rho binds nascent RNA first and catches up with paused RNA polymerase (RNAP) and this catch-up Rho mediates simultaneous releases of transcript RNA and template DNA from RNAP. The fastest route is that the catch-up Rho effects RNA-only release and leads to 1D recycling of RNAP on DNA. The slowest route is that the RNAP-prebound stand-by Rho facilitates only the simultaneous rather than sequential releases. Among the three routes, only the stand-by Rho's termination efficiency positively correlates with pause duration, contrary to a long-standing speculation, invariably in the absence or presence of NusA/NusG factors, competitor RNAs or a crowding agent. Accordingly, the essential terminational pause does not need to be long for the catch-up Rho's terminations, and long pauses benefit only the stand-by Rho's terminations. Furthermore, the Rho-dependent termination of *mgtA* and *ribB* riboswitches is controlled mainly by modulation of the stand-by rather than catch-up termination.

## Graphical Abstract

**Graphical Abstract:**
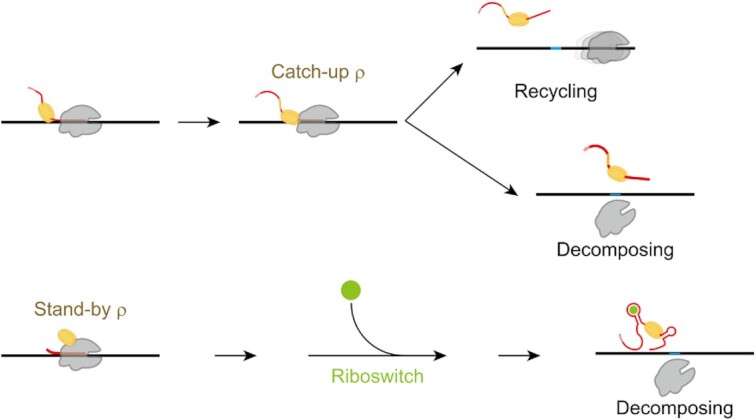
Among the three compatible routes of ρ-dependent termination, riboswitch gating regulates the slowest route of the stand-by decomposing termination more predominantly than the two faster routes by the catch-up ρ.

## INTRODUCTION

Transcriptional pauses play critical roles for gene regulation in all stages of transcription. In bacteria and archaea, for example, the pauses provide time windows for RNA polymerase (RNAP) to bind transcription factors (1,2) and coordinate transcription-translation coupling (3,4). Similarly in eukaryotes, the pauses keep promoters accessible to transcription factors by blocking nucleosome reassembly and are involved in various co-transcriptional processes (5,6). Pausing is also important for appropriate folding of diverse regulatory RNAs. For instance, RNA folding of riboswitches, which regulate transcription termination (7–9), translation initiation (10–14) or RNA stability (10,15–18), is often controlled by the pause.

Specifically, transcriptional pause is considered essential for efficient termination, as it provides a time for stable elongation complexes (ECs) to undergo the conformational changes necessary for releasing transcript RNA at termination. The eukaryotic RNAP II pausing beyond polyadenylation sites facilitates terminational breaking up with left-over RNAs (19–21). In bacteria, RNAP pausing is caused by oligo(U) sequences to secure enough time for terminator hairpin formation in the intrinsic, RNA hairpin-dependent termination (22). Pausing is also requisite in the bacterial extrinsic, Rho (ρ) factor-dependent termination (23) and a pause-defective RNAP variant is resistant to ρ (24).

In the primary pre-terminational mechanism of ρ-dependent termination, ρ binds nascent RNA first at a *rut* (Rho utilization) site and moves down along the RNA using ATP hydrolysis energy to catch up with the RNAP that is pausing at a downstream termination site. In this catch-up termination, a.k.a. tracking (25), kinetic-coupling (26), RNA-dependent (27) or RNA-centric model (24), RNAP pausing should be long enough for the catching-up ρ to approach the pausing site where termination takes place. In the secondary mechanism, ρ pre-binds the RNAP that has not yet reached the terminational pausing site, and stands by for an incipient RNA *rut* site to freshly emerge out of the RNAP. In this stand-by termination, previously called allosteric (28), RNAP-dependent (27) or EC-centric model (24), the standing-by ρ has been assumed not to need extra time to bind the *rut* site and trigger termination at the pausing site.

The two long-confronting pre-terminational mechanisms by the RNA-bound catch-up ρ and the RNAP-prebound stand-by ρ were recently revealed in our study to operate compatibly together in any single terminator (29). As to their post-terminational outcomes, the catch-up ρ’s termination results in (i) concomitant releases of RNA and DNA from RNAP for one-step decomposition of EC via the catch-up decomposing route, or (ii) delayed release of DNA long after RNA release from RNAP for 1D recycling of the post-terminational RNAP diffusing on DNA via the catch-up recycling route, while (iii) the stand-by ρ’s termination leads only to the decomposing outcome via the stand-by decomposing route.

In addition to disclosing the three routes' co-existence, our previous study unveiled that they operate on different timescales (29). The catch-up recycling route is the fastest and the stand-by decomposing route is the slowest among the three routes. This chronological order is directly opposite to the above-mentioned speculation. This raises an important question about the role of transcriptional pause in the three routes.

To address the question in this study, we measured the pause durations (*t*_p_) and the individual route termination efficiencies (TEs) of several ρ-dependent terminators using the single-molecule assays that we lately developed (29), and examined the pause-termination correlations between *t*_p_ and TEs. The correlation is invariably positive for the stand-by decomposing termination rather than the catch-up decomposing or recycling termination. Furthermore, we measured ligand dependency of TEs with two riboswitch-regulated ρ-dependent terminators, and discern that the riboswitches regulate overall TEs mainly by modulating the stand-by decomposing termination.

## MATERIALS AND METHODS

### Preparation of proteins and total RNA

*Escherichia coli* RNAP core enzyme (30) and RpoD, a.k.a. σ^70^, (31) were separately purified as previously described, or the holoenzyme was purchased from New England Biolabs. The wild-type *E. coli* ρ was purchased from Bioprogen. The mutant ρ^P279S^ was prepared as previously described (29,32) using its expression plasmid provided by Dr Ranjan Sen in Hyderabad, India. Total RNA was prepared from *E. coli* BL21(DE3) using the EZ^TM^ Total RNA Miniprep Kit purchased from Enzynomics.

### Preparation of transcription templates

Using the DNA oligomers (Supplementary Table S1) purchased from Integrated DNA Technologies, the nontemplate DNA strands were prepared by annealing their two parts with a DNA splint. They were slowly cooled from 90°C to 16°C and ligated using the T4 DNA ligase 2 purchased from New England Biolabs in a buffer of 50 mM Tris–HCl, pH 8.0, 10 mM MgCl_2_, 10 mM dithiothreitol and 1 mM ATP. Double-stranded DNAs were made by polymerase chain reactions using the 5'-biotin-labeled forward primers and the 5'-Cy5-labeled reverse primers.

### Single-molecule fluorescence experiments

Stalled ECs were prepared by incubation of the biotinylated Cy5-DNA with 250 μM 5'-Cy3-ApU (TriLink BioTechnologies), 10 μM ATP (GE Healthcare), 10 μM CTP (GE Healthcare) and 340 nM RNAP holoenzyme with σ^70^ in a transcription stalling buffer of 20 mM Tris–HCl, pH 8.0, 20 mM MgCl_2,_ 20 mM NaCl and 1 mM dithiothreitol for 20 min. They were placed in a chamber sandwiched between the quartz slide and the cover slip that were both thoroughly cleaned and coated with a 40:1 mixture of polyethylene glycol (PEG) and biotinylated PEG (Laysan Bio Korea). Consequently, they were immobilized on both surfaces in a scattered way via biotin-streptavidin-biotin conjugation (33).

Fluorescence images were obtained from the samples immobilized on the quartz slide rather than the cover slip using a home-build prism-type total internal reflection fluorescence microscope with a 1-s exposure time in the alternating laser excitation mode (34) so the time resolution was 2 s. Transcription assays were performed at 37°C in a buffer of 40 mM Tris–HCl, pH 8.0, 10 mM MgCl_2,_ 150 mM KCl and 1 mM dithiothreitol, while the buffer was supplemented with 5 mM protocatechuate acid and 100 nM protocatechuate-3,4-dioxygenase to reduce photobleaching of the fluorophores (35) and with saturated Trolox to suppress their blinking (36). When needed, 100 nM wild-type or mutant ρ hexamer, 500 nM NusA plus 500 nM NusG, 1 mg/l total RNA from *E. coli* and 5% PEG-8000 (New England Biolabs) were added, and the MgCl_2_ and ribocil-C (MedChemExpress) concentrations were varied as indicated. After washing, transcription was resumed by adding an NTP mixture (200 μM each) to the chamber using a syringe pump Fusion 100 (Chemyx).

Single-molecule images were taken while both Cy3 and Cy5 were alternately excited by a 532-nm green laser Excelsior-532-50-CDRH and a 640-nm red laser Excelsior-640c-60-CDRH (Spectra-Physics), respectively. The fluorescence signals were collected by a water-immersion objective lens UPlanSApo 603 (Olympus) and filtered through a 532-nm notch filter NF03-532 E-25 and a 632-nm notch filter NF03-633 E-25 (Semrock) to reject scattered laser lines. They were then separated with a dichroic mirror 635dcxr (Chroma) before imaged onto an electron-multiplying charge-coupled device camera iXon DU-897 (Andor Technology). Software IDL 7.0 (ITT), MATLAB R2018a (MathWorks), Excel 2016 (Microsoft) and Origin 8.5 (OriginLab) were used for data analyses.

### Statistical analyses

The Pearson's *r* was estimated for linear correlation between *t*_p_ and ρ-dependent TE using Origin 8.5. Tested additionally was a null hypothesis that the linear fitting slope is zero, and calculated was probability *P* of the null hypothesis using the formula below.


$$\begin{equation*}P\ = {\rm{\ }}1 - {t}_{{N} - 2}\left( {\frac{{\left| {{b}_1} \right|}}{{SE}}} \right)\end{equation*}$$

where *t*_*N*_ denotes the Student's t-distribution whose degree of freedom is *N*, *b*_1_ denotes the fitting value of the slope, and *SE* denotes the standard error of fitting value. All the reported *P* values are one-sided and statistical significance is defined as *P* ≤ 0.05.

### Time-course bulk transcription assays

*E. coli* holoenzyme (30 nM) and a template DNA (15 nM) with the wild-type or a mutant of *mgtA* were incubated with or without *E. coli* ρ hexamer (100 nM) in a transcription buffer of 40 mM Tris–HCl, pH 8.0, 200 μM ATP, 200 μM GTP, 200 μM UTP, 25 μM CTP, 0.75 μCi of [α-^32^P]CTP (3000 Ci/mmol), 10 mM MgCl_2_, 150 mM KCl and 2 mM dithiothreitol at 37°C for varying reaction time. The reaction was quenched by addition of a 2× urea-denaturing gel loading buffer and analyzed on a 6% polyacrylamide–7 M urea gel.

## RESULTS

### Transcription termination and readthrough are monitored at the single-molecule level

**Figure 1.**
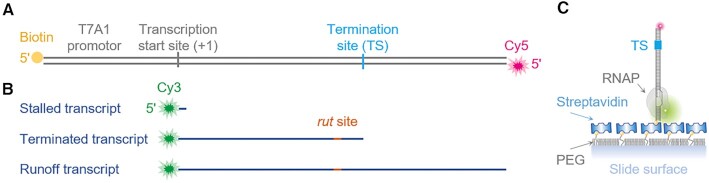
Fluorescent transcription complexes for single-molecule monitoring. (**A**) An exemplary fluorescent DNA template containing a single *E. coli* transcription unit with a ρ-dependent terminator. The template (gray) is labeled with a red-fluorescent Cy5 (magenta) at the downstream end for its real-time monitoring as well as with biotin (yellow) at the upstream end for its surface immobilization. (**B**) Its transcripts (blue) that are produced to have a greenish-yellow-fluorescent Cy3 (green) at the 5' end. When transcription elongation is resumed from the stalled transcripts, either termination occurs at the termination site (TS) to produce the terminated transcripts or readthrough takes place to yield the runoff transcripts. Both product transcripts have a *rut* site (orange) that ρ (not shown) can bind. (**C**) A fluorescent EC tied down on a microscope slide. A complex of RNAP, DNA and RNA with or without ρ is fixed on the polyethylene glycol (PEG)-coated surface through biotin-streptavidin-biotin conjugation. Individual complexes are spatially dispersed on the surface using a fraction of the biotin moieties each attached to only one in 41 PEG molecules.

Among the several single-molecule assays that we established to explore the intrinsic and ρ-dependent termination mechanisms by *E. coli* RNAP (29,37,38), three assays were performed in this study on assorted ρ-dependent terminators, and their experimental schemes and representative results are briefed here. DNA templates each contain the T7A1 promoter and a ρ-dependent terminator, and are each labeled with biotin at the upstream end for surface immobilization and fluorescently with reddish Cy5 at the downstream end for individual real-time monitoring (Figure 1A).

Stalled ECs are prepared to be additionally fluorescent with greenish-yellow Cy3 at transcript RNA. The Cy5-DNA is incubated with 5'-Cy3-ApU dinucleotide, ATP, CTP and *E. coli* RNAP holoenzyme with σ^70^ in a transcription stalling buffer. Transcription starts preferentially with ApU, so nascent RNAs get a Cy3 label at the 5' end (Figure 1B). Because GTP and UTP are missing in the buffer, transcription stalls with an RNA that is 4–8 nucleotides (nt) long depending on the templates. The stalled ECs are fixed dispersedly on microscope slides via biotin-streptavidin conjugation (Figure 1C) and thoroughly washed for removal of the unbound before subjected to fluorescence imaging.

The Cy3 exhibits protein-induced fluorescence enhancement (PIFE) when the 5' Cy3-end of RNA is still proximal to RNAP at the stalling site, as the protein binding restricts the cyanine dye's photoisomerization from fluorescent *trans*-isomer to non-fluorescent *cis*-isomer and intensifies its fluorescence (39). The Cy3 PIFE diminishes when four NTPs are injected to resume the stalled transcription so the transcript grows in length and its 5’ end thereby moves away from the transcribing RNAP. This stepwise decline of the Cy3 signal identifies active ECs. Finally, the Cy3 signal itself disappears when the Cy3-transcript is released from EC at termination and diffuses away from its spot long before photobleaching destroys Cy3.

The Cy5 also displays PIFE when its photoisomerization is hindered by the RNAP contacting the downstream Cy5-end of DNA in two instances. One is when transcription readthrough takes place at the termination site and the continuously transcribing RNAP comes to the end for runoff transcription. It is here called readthrough runoff. The other instance is when termination occurs with RNA-only release and the 1D recycling RNAP diffuses on DNA to the end, i.e. after the recycling termination. No PIFE is shown if RNAP with or without RNA falls off DNA before arriving at the end.

**Figure 2.**
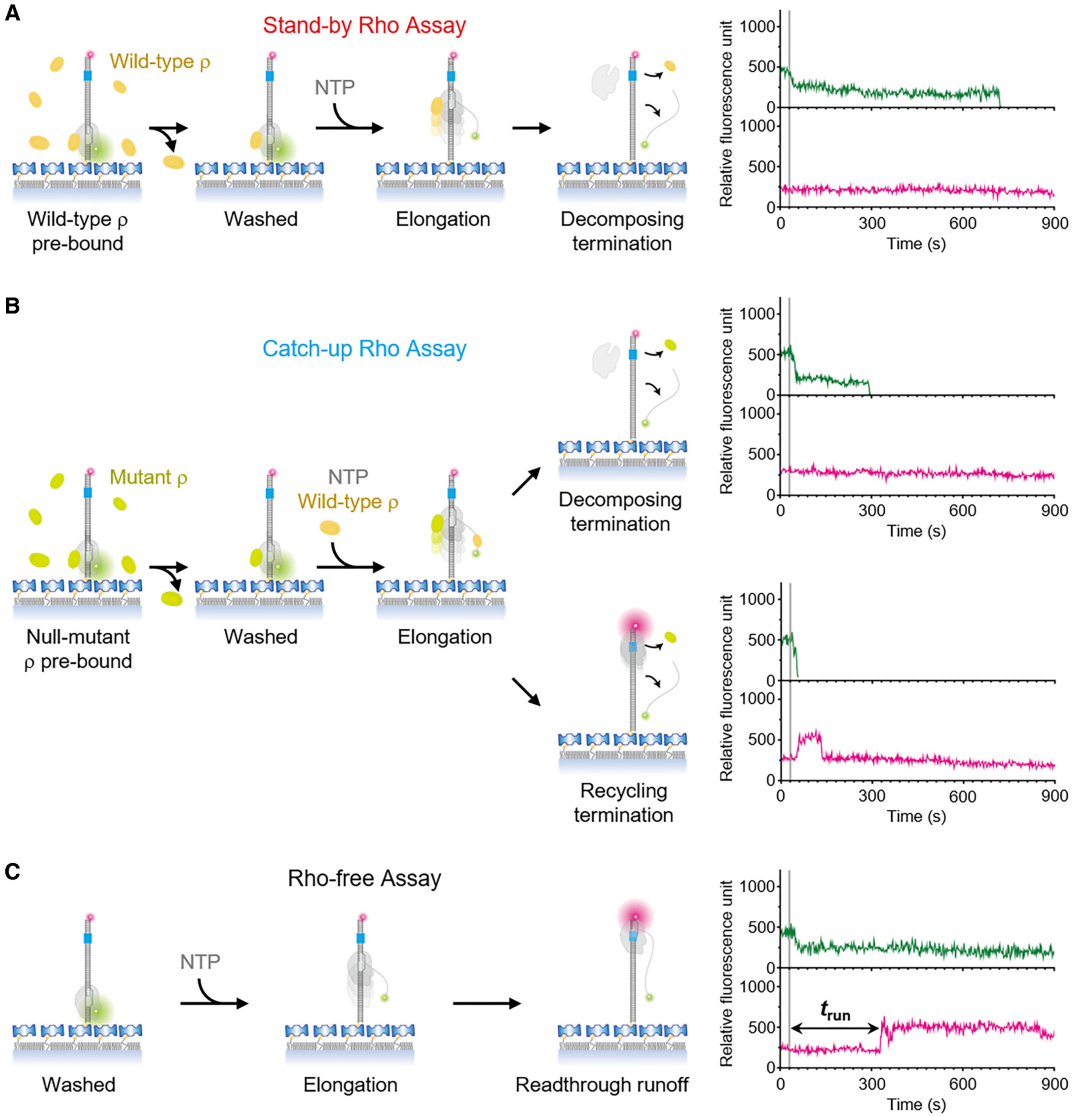
The three single-molecule assays with ρ-dependent terminators. (**A**) Left panel: the stand-by ρ assay's experimental scheme. PIFE occurrence of Cy3 (green) or Cy5 (magenta) is indicated by an enlarged circle. Right panel: the representative fluorescence time traces at Cy3 (top) and Cy5 (bottom) excitations exemplifying the stand-by decomposing termination route. NTPs were injected at 30 s (gray vertical line). (**B**) Left panel: the catch-up ρ assay's scheme. Right panel: the traces illustrating the catch-up decomposing termination route (top) and the catch-up recycling termination route (bottom). (**C**) Left panel: the ρ-free assay's scheme. Right panel: the traces demonstrating the readthrough runoff transcription. The delay between Cy3 PIFE diminishing and Cy5 PIFE appearance, denoted by *t*_run_, corresponds to the elongation timespan without ρ.

In the stand-by ρ assays (Figure 2A), we pre-incubate the stalled ECs with ρ to make up stable ρ·RNAP complexes, wash out the unbound ρ and resume transcription without additional ρ. Only the stand-by rather than catch-up ρ’s termination is monitored and all ECs decompose in one step with synchronous releases of RNA and RNAP from the immobilized DNA as we recently observed (29). This decomposing termination is characterized by Cy3 signal disappearance without Cy5 PIFE occurrence, while the readthrough runoff is specified by that Cy5 PIFE appears with the transcribing RNAP nearing the downstream Cy5-end after Cy3 PIFE diminishes upon elongation resumption.

In the catch-up ρ assays (Figure 2B), by contrast, we pre-incubate the stalled ECs with a completely inactive mutant ρ^P279S^ (29,32) instead of the wild-type to pre-occupy the ρ-binding site(s) of RNAP, wash out the unbound mutant and resume transcription along with the wild-type ρ. The pre-bound null-mutant ρ cannot mediate the stand-by termination and is hardly replaced by the subsequently added wild-type ρ (29), so only the catch-up ρ-dependent termination occurs. With the catch-up ρ, both decomposing and recycling outcomes are expected as we have previously observed (29). The recycling route manifests Cy5 PIFE after Cy3 disappearance unlike the decomposing route without Cy5 PIFE occurrence.

In each assay, every active EC spot is counted as either readthrough runoff, decomposing termination or recycling termination event depending on its Cy3 and Cy5 signal patterns. Their relative frequencies yield the decomposing, recycling and overall TEs of stand-by or catch-up ρ. In order to give the ρ-dependent TEs (ρTEs) tabulated in Supplementary Table S2, from these raw measurements of TEs subtracted are the background TEs measured in the ρ-free assays (Figure 2C), where we resume the stalled transcription without ρ at all. Therein transcription reads through the termination site and runs off the downstream end, except for a low level of ρ-independent background termination leading to the decomposing outcome via the ρ-free decomposing route (29).

### Pause duration correlates with the stand-by, but not catch-up, termination efficiency

Transcriptional pause at the termination sites is required for both catch-up and stand-by terminations like all other types of termination and here called terminational pause. Additionally, it has been presumed that RNAP pauses at the termination sites long enough to be caught by the catch-up ρ, whereas the stand-by ρ already pre-bound to RNAP does not need such extra length of pause for its action. It has been recently found, however, that the stand-by terminations usually occur later than the catch-up terminations (29). Thereupon, we examined whether and how the three routes are differently affected by the pauses.

The terminational pause durations cannot be directly measured but can be approximated in our experimental settings. We then examined the correlations of *t*_p_ with ρTEs of the three routes measured in the two ρ assays. Firstly employed in this study were the same DNA templates with the *mgtA*, *rho*, *ribB*, *trp-t’* or *tR1* ρ-dependent terminator as we have previously confirmed their termination proficiencies and characteristics using both single-molecule and bulk transcription assays (29), but overall 2.7-fold more ECs are analyzed to further reduce error bars in this study (*n* = 34 741) than the previous one (*n* = 12 838).

In order to estimate the *t*_p_ values, we measured the ρ-free elongation timespans *t*_run_ that transcribing RNAP takes to run from the stalling site to the downstream Cy5-end (Figure 2C). RNAP’s reaching the end is timed by the start of Cy5 PIFE since the NTP injection for resumption of the stalled transcription in the ρ-free assays. Each *t*_run_ value hence includes the timespan *t*_inc_ of NTP incorporation and the timespan *t*_p_ of all possible pauses occurring in the entire stalling site-downstream region. As *t*_inc_ can be approximated using the average speed of NTP incorporation measured without ρ, *t*_p_ was then estimated by *t*_p_ = *t*_run_ – *t*_inc_. The *t*_run_ can be additionally obtained in the two ρ assays, but *t*_p_ values were not calculated from those, because it is not known whether and how much *t*_inc_ is changed by the presence of ρ.

**Figure 3.**
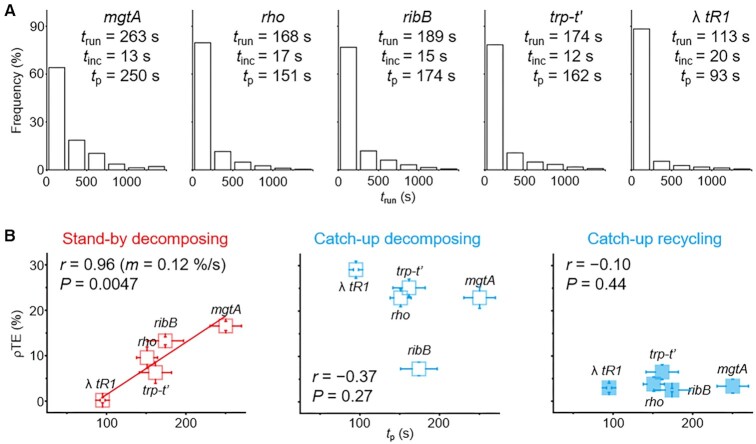
Measurements of pause and termination with ρ-dependent terminator templates. (**A**) Pause durations (*t*_p_) of the five terminator templates. After the ρ-free elongation timespans (*t*_run_) were separately measured with the *mgtA* (*n* = 387), *rho* (*n* = 1849), *ribB* (*n* = 1202), *trp-t’* (*n* = 2409) and λ *tR1* (*n* = 335) terminator templates and the NTP incorporation timespan (*t*_inc_) was estimated for each template, their pause durations were calculated by *t*_p_ = *t*_run_ – *t*_inc_. (**B**) Correlation between *t*_p_ and ρTE. The ρTEs of the stand-by decomposing (left), catch-up decomposing (middle) and catch-up recycling (right) termination routes were separately measured and are plotted on the y-axis against *t*_p_ on the x-axis. The Pearson's *r*, *P*-value and the fitting line slope (*m*) are shown. Error bar represents standard deviation of three independent datasets. The numbers of analyzed molecules are in Supplementary Table S2.

The *E. coli* NTP incorporation speed without ρ has previously ranged from 10 to 30 nt/s under the conditions similar to ours with 200 μM NTP each (40–43). When its midrange speed 20 nt/s is used to divide the runoff transcript length, an approximate *t*_inc_ is estimated as the minimum elongation timespan without any pause, and varies between 12 (*trp-t'*) and 20 s (*tR1*) with the five terminator templates (Figure 3A). Contrastingly, the measured *t*_run_ values span from 113 (*tR1*) to 263 s (*mgtA*) and are an order of magnitude larger than the *t*_inc_ values, indicating that the elongation on each template is much retarded by lengthy or abundant pausing. Then, *t*_p_ is diverse from 93 (*tR1*) to 250 s (*mgtA*), or 1.6 to 4.2 min, which is a range of 2.7-fold difference.

Pauses take place not only at single or multiple termination sites but also possibly other sites, so *t*_p_ is the sum total of all the pause durations. However, the contribution of non-terminational pauses to *t*_p_ estimation appears negligible on the *mgtA* template and its eight mutants described below, according to the time-course bulk transcription assays (Supplementary Figure S1). Then, we investigated how *t*_p_ relates to ρTEs of the three routes with each terminator (Figure 3B). The riboswitch-associated terminators *mgtA* and *ribB* were under their *rut* site-exposing conditions respectively with 10 mM Mg^2+^ and 200 nM ribocil-C to be compared to the other three terminators without a riboswitch.

Among the three routes, only the stand-by decomposing ρTE shows a significantly positive linear correlation with *t*_p_ as the Pearson correlation coefficient *r* = 0.96, close to one, whereas *r* = −0.37 and −0.10 for the catch-up decomposing and recycling routes, respectively. Additionally, only the stand-by decomposing termination data exhibit *P* = 0.0047, lower than a significance threshold α = 0.05, while *P* = 0.27 and 0.44 respectively for the other two routes. Thus, *t*_p_ positively correlates with the stand-by but not catch-up ρTEs. Even when different speeds of NTP incorporation are used, affected are *t*_p_ estimates but not their correlation strengths with any ρTE (Supplementary Figure S2).

**Figure 4.**
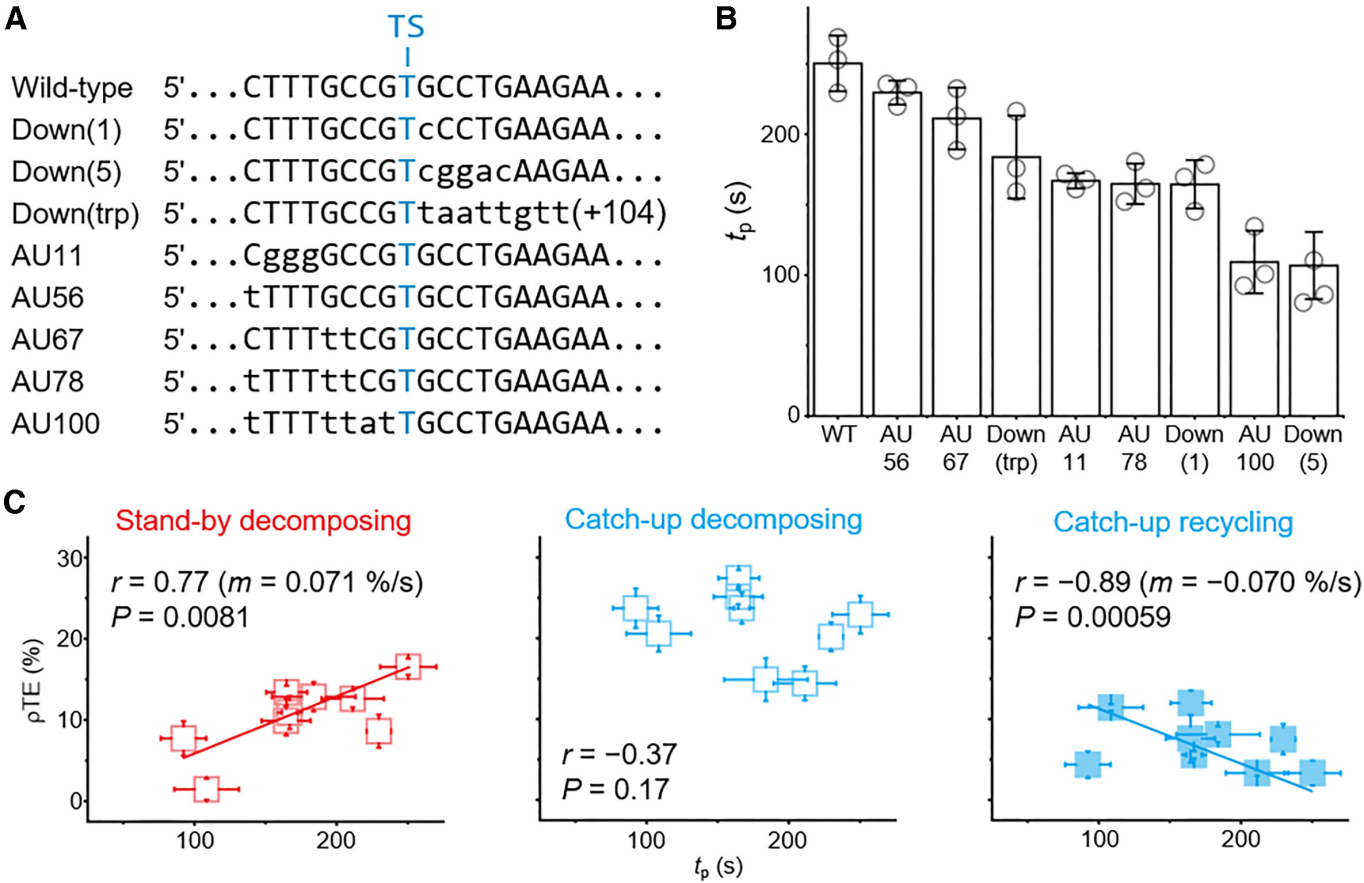
Mutational analysis of the pause-termination correlations. (**A**) The eight mutations introduced to the *mgtA* terminator template around the major termination site (TS). The varied sequences are in lower cases. (**B**) The *t*_p_ values of the *mgtA* wild-type (WT) and mutant templates estimated as described in Figure 3A. (**C**) The correlation between *t*_p_ and ρTE analyzed as explained in Figure 3B. Error bar represents standard deviation of three independent datasets. The numbers of analyzed molecules are in Supplementary Table S2.

Next, a total of eight mutations (Figure 4A) were introduced in the *mgtA* terminator template that has a single major terminational pause site (7,23,29) and the longest *t*_p_ among the five terminator templates (Figure 3A). Three mutations are located downstream of the major termination site, and the other five are upstream but within the region that would form an RNA·DNA hybrid on the pausing at the major termination site. In the Down(trp) mutant, the entire termination site-downstream region is replaced by the *trp-t'* downstream sequence. Thus, the variations include the template region that would be encapsulated by the RNAP paused at the major termination site, which is not shifted by any of these mutations (Supplementary Figure S1).

The mutants' *t*_p_ values fluctuate between 107 and 230 s, or 1.8 and 3.8 min (Figure 4B and Supplementary Figure S3). They are all shorter than the wild-type *t*_p_ of 250 s and range similarly to the wild-type terminators, while their background TEs are similarly as low as 5.3–8.4% (Supplementary Figure S4A). All these mutations modify the immediate vicinity of the major termination site, and their shortening of *t*_p_ comes from the terminational pause site more likely than the non-terminational ones (Supplementary Figure S1). The *t*_p_-ρTE correlation (Figure 4C) is positively linear for the stand-by decomposing (*r* = 0.77, *P* = 0.0081) and little significant for the catch-up decomposing (*r* = −0.37, *P* = 0.17) but negatively linear for the catch-up recycling (*r* = −0.89, *P* = 0.00059).

The negative linearity for the catch-up recycling is not observed with the five wild-type terminators as described above (Figure 3B) or the three termination-site-downstream mutants of *mgtA* (Supplementary Figure S5A). However, it is significant in the five hybrid-region mutants (Supplementary Figure S5B) as the AU content of the termination-site hybrid region correlates with both *t*_p_ and ρTE (Supplementary Figures S5C and D, respectively). Thus, the negative linearity can be ascribed to the AU content variation, which correlates with proficiency of the RNA shearing mechanism that facilitates recycling termination as we previously observed (29).

In conclusion, both wild-type and mutant terminator data consistently indicate that the terminational pause duration has a positively linear correlation with the stand-by decomposing ρTE. The positive correlation does not generally hold with catch-up decomposing or recycling ρTE.

### Slow recognition of RNA *rut* site by the stand-by ρ permits the riboswitch regulations

As just explained, the stand-by decomposing termination is more efficient with longer pauses than shorter ones. Besides, it occurs definitely later than the catch-up recycling termination (Supplementary Figure S6 and Supplementary Table S3) despite that the stand-by ρ binds RNAP earlier than the catch-up ρ. This slow route for the stand-by decomposing termination is found in this study to play a predominant role in the regulatory operation of riboswitches for the ρ-dependent terminators associated with them.

**Figure 5.**
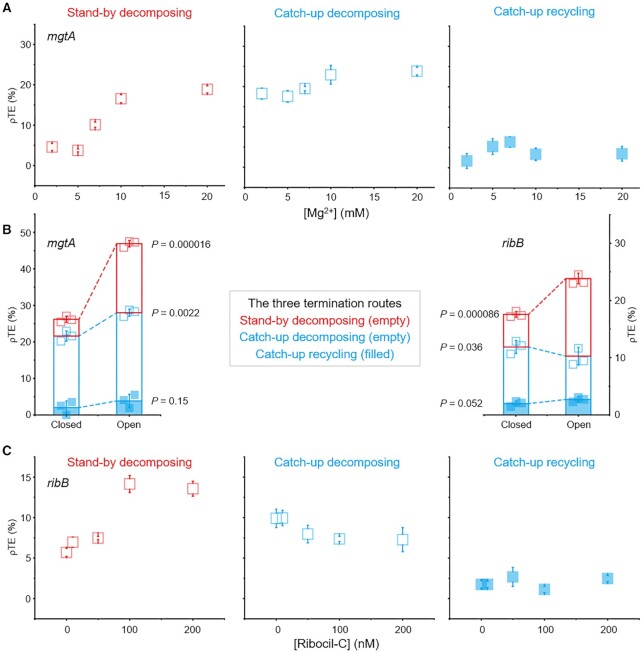
Riboswitch ligand dependence of the three ρ-dependent termination route efficiencies. (**A**) Dependency of individual route's ρTE of the *mgtA* terminator on its riboswitch ligand Mg^2+^. The ρTEs of the stand-by decomposing (left graph), catch-up decomposing (middle graph) or catch-up recycling (right graph) route of *mgtA* on the y-axis are plotted against Mg^2+^ concentrations on the x-axis. Error bar represents standard deviation of three independent datasets. (**B**) Direct comparison of the individual route and total ρTEs between the riboswitch mostly closed at 2 mM Mg^2+^ or no ribocil-C and the one mostly open at 20 mM Mg^2+^ or 200 nM ribocil-C. (**C**) Plots of the *ribB* route ρTEs on the y-axis against ribocil-C concentrations on the x-axis similarly to Figure 5A. The numbers of analyzed molecules are in Supplementary Table S2.

A magnesium ion-sensing riboswitch is encoded by the *Salmonella mgtA* gene's leader region and can adopt alternative RNA conformations to regulate its ρ-dependent terminator (7,9,23). At high Mg^2+^ levels, the *mgtA* terminator *rut* site is exposed or open to allow for efficient termination at the leader's downstream end to preempt the transcription of its downstream *mgtA* gene encoding a Mg^2+^-importing ATPase. At low Mg^2+^ levels, the *rut* site is hidden or closed in a stem-loop structure to prevent the termination to allow for the *mgtA* transcription leading to an increased import of Mg^2+^ by the transporter. We investigated how the three route efficiencies of *mgtA* terminator are individually augmented by increasing Mg^2+^ concentration (Figure 5A).

Opening of the *mgtA rut* site by increasing Mg^2+^ from 2 to 20 mM raises the stand-by decomposing ρTE by the most among the three routes, by 14.3 percentage points (pp), while only 5.9 and 0.9 pp rise in the catch-up decomposing and recycling ρTEs, respectively (Figure 5B, left panel). Thus, the stand-by route contributes 68% to the total termination increment by the Mg^2+^ elevation, which gates the *mgtA* riboswitch and terminator. Apparently, the stand-by ρ pre-bound to RNAP is more sensitive to the structural changes of the *rut*-harboring RNA than the catch-up ρ free in solution.

Another riboswitch-controlled ρ-dependent terminator is present just upstream of the *E. coli ribB* gene participating in the biosynthesis of riboflavin (7,9). The *ribB* leader's terminator contains a flavin mononucleotide (FMN)-sensing riboswitch. With abundant FMN, the *ribB rut* site is open to allow for efficient termination to attenuate the downstream *ribB* expression and riboflavin synthesis. With scarce FMN, the *rut* site is closed in a hairpin structure to impede the termination and facilitate the *ribB* transcription. Because the greenish-yellow fluorescence of FMN interferes with the single-molecule imaging of Cy3, we used non-fluorescent ribocil-C in place of FMN as a riboswitch ligand (44,45) to inquire how the three route efficiencies of *ribB* terminator are affected by ribocil-C variation (Figure 5C).

When the *ribB rut* site's open state with 200 nM ribocil-C is compared to its closed state without ribocil-C, the stand-by decomposing ρTE is 7.0 pp higher, whereas the catch-up decomposing ρTE is 3.0 pp lower and the catch-up recycling ρTE is only 0.9 pp higher (Figure 5B, right panel). Thus, the *ribB* riboswitch opening much increases the stand-by decomposing termination, but even decreases the catch-up decomposing termination, which is an opposite but minor effect. Although it remains to be verified whether FMN has the same effects as ribocil-C, the riboswitch RNA conformation could be more favorable in its presence than absence for the stand-by ρ to find a *rut* site and mediate the decomposing termination but less favorable for the catch-up ρ to do so.

While the background TEs of *mgtA* or *ribB* terminator template are 6.1 to 16% at the ligand concentrations varied in the ρ-free assays (Supplementary Figures S4B and C, respectively), the stand-by decomposing route is modulated more than the catch-up decomposing or recycling route in both terminators. Thus, the stand-by ρ seems more sensitive to the riboswitch's conformational changes that open or close their *rut* sites than the catch-up ρ. Despite that the catch-up decomposing ρTE could be adversely affected by the *ribB* riboswitch, dominantly attuned is the stand-by ρTE in both *mgtA* and *ribB* terminators.

### Trivial effects of NusA/NusG, competitor RNAs and a crowder on the pause-termination correlations

All the above experiments were performed with a minimal set of transcription complex components including core RNAP, σ and ρ but without any other associating factors. We then carried out three auxiliary sets of experiments each with an addendum that may affect transcriptional pause and termination. Firstly, NusA and NusG factors are known to bind RNAP and affect its pause, termination or both in one way or another (24,46). Thus, we repeated the measurements of *t*_p_ and ρTE in the five wild-type terminator templates with addition of *E. coli* NusA and NusG together (NusA/G) in order to see if the addendum modifies the *t*_p_-ρTE correlations.

Secondly, ρ binds RNA not only specifically at the *rut* sites but also nonspecifically at other sites. The nonspecific binding could produce a sponge effect to reduce the effective concentration of ρ for the specific binding (47). As it has not been reported whether and how much the sponge effect would be exerted on the pause and termination, we additionally measured *t*_p_ and ρTE in the five terminator templates with inclusion of *E. coli* total RNA mostly as nonspecific competitors and examined the pause-termination correlations.

Thirdly, the intracellular environment is highly crowded with macromolecules, and the transcription initiation rate has been reported to be accelerated by crowding compounds (48), although their effects on the elongation, pause or termination are not known yet. For example, large crowders such as PEG-8000 increase both viscosity and transcription kinetics (48). Thus, we examined the macromolecular crowding effects of PEG-8000 on the pause, termination and their correlations.

NusA/G or total RNA increase *t*_p_ in the *tR1* and *trp-t'* terminators but not much in the *mgtA*, *rho* or *ribB* terminator, while PEG-8000 raises *t*_p_ in all the five terminators (Supplementary Figure S7A). Their effects on ρTE vary greatly depending on the terminators (Supplementary Figure S7B). Noteworthily, the stand-by termination is virtually null without NusA/G, total RNA and PEG-8000 but becomes a major or substantial route with either of them in the *tR1* terminator, whereas it is downgraded by them in the *rho* and *ribB* terminators. With any of the addendums, the overall ρTE either decreases or maintains rather than increases in any terminator, while the addendum's effects on the individual route efficiencies depend on the addendum and the terminator.

**Figure 6.**
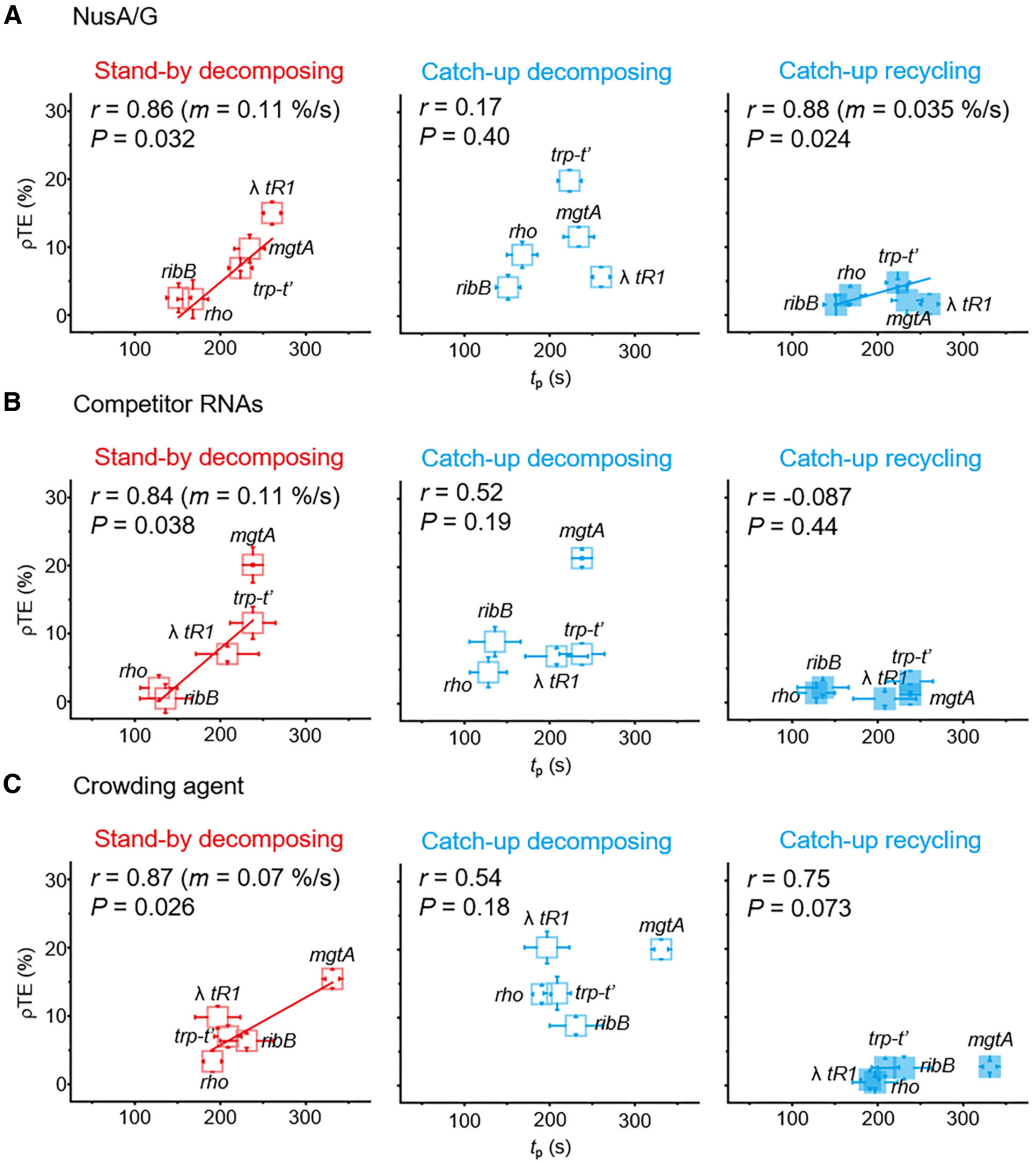
The pause-termination correlations in the presence of NusA/G, competitor RNAs or a crowder. The *t*_p_ and ρTE values of the five terminator templates were measured with addition of *E. coli* NusA/G factors (**A**), *E. coli* total RNA (**B**) or PEG-8000 (**C**). The recycling (solid) and decomposing (open) terminations were counted with each terminator in the stand-by (red) and catch-up (cyan) ρ assays. The ρTEs of the stand-by decomposing (left), catch-up decomposing (middle) or catch-up recycling (right) termination route are separately plotted on the y-axis against *t*_p_ on the x-axis. The Pearson's *r*, *P*-value and the fitting line slope (*m*) are shown. Error bar represents standard deviation of three independent datasets.The numbers of analyzed molecules are in Supplementary Table S2.

With NusA/G, the linear *t*_p_-ρTE correlation is highly positive for the stand-by decomposing (*r* = 0.86, *P* = 0.032, the slope *m* = 0.11%/s), little significant for the catch-up decomposing (*r* = 0.17, *P* = 0.40) and less positive for the catch-up recycling (*r* = 0.88, *P* = 0.024, *m* = 0.035%/s) as shown in Figure 6A. With total RNA, the correlation (Figure 6B) is positive for the stand-by decomposing (*r* = 0.84, *P* = 0.038, *m* = 0.11%/s) but barely significant for the catch-up decomposing (*r* = 0.52, *P* = 0.19) or recycling (*r* = −0.087, *P* = 0.44). Similarly, with PEG-8000, the positive linear correlation (Figure 6C) is significant for the stand-by decomposing (*r* = 0.87, *P* = 0.026, *m* = 0.07%/s) but not for the catch-up decomposing (*r* = 0.54, *P* = 0.18) or recycling (*r* = 0.75, *P* = 0.073).

Thus, both *t*_p_ and ρTE are distinctly altered in individual terminators by the inclusion of NusA/G, competitor RNAs or a crowder. After all, with either of them, the linear *t*_p_-ρTE correlations remain qualitatively the same for the slow decomposing termination routes; still much positive for the stand-by decomposing route and poor for the catch-up decomposing route. On the other hand, the correlation is poor or only slightly positive for the fastest, catch-up recycling termination route.

## DISCUSSION

We have recently unveiled the three coexisting but kinetically different routes of ρ-dependent termination (29). Here, we uncover that among the three routes, only the stand-by ρ’s sole route for decomposing outcome, the slowest route, is invariably profited by pause prolongation as this termination becomes more efficient with longer pauses. In contrast, the catch-up ρ’s major route for decomposing outcome, the most frequent route, is hardly affected by pause lengthening or shortening as its ρTE little correlates with *t*_p_. Meanwhile, the catch-up ρ’s minor route for recycling outcome, the fastest route, is either unaffected or affected positively or negatively by pause elongation depending on the situation.

**Figure 7.**
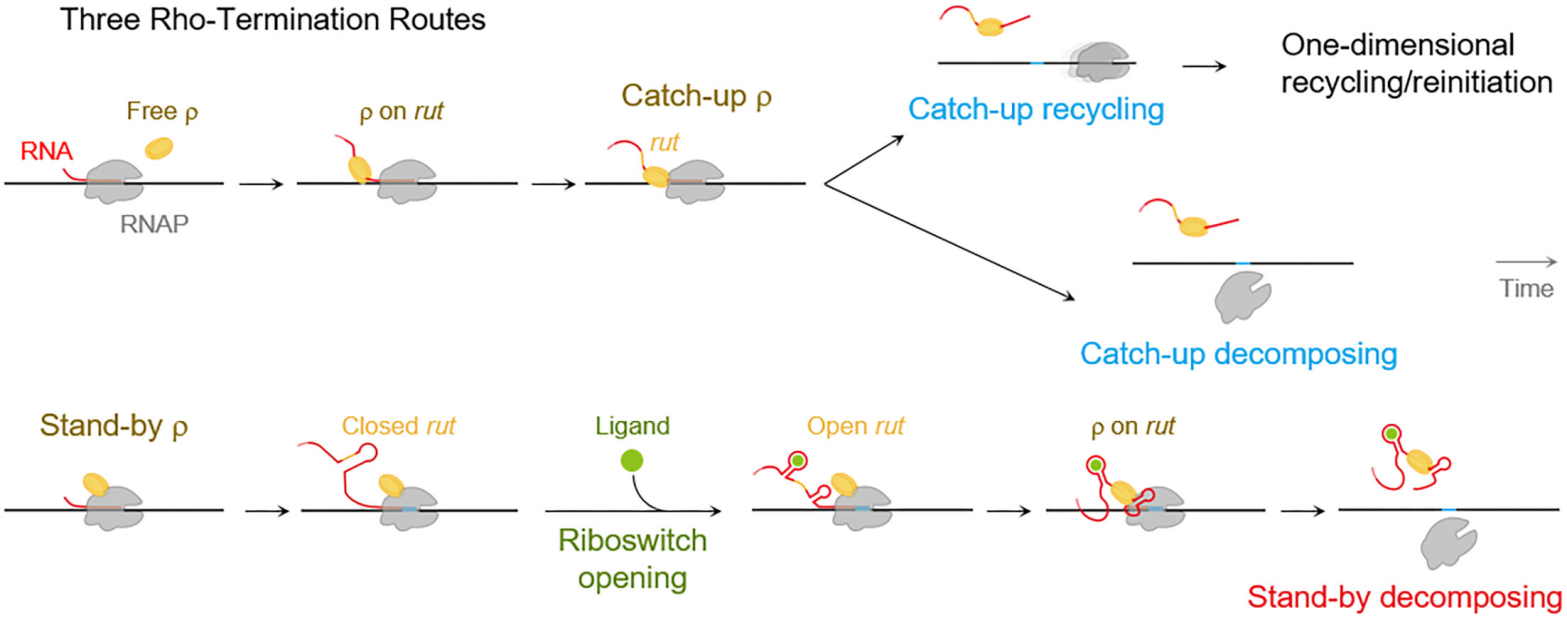
Weighted effects of riboswitch gating on the three compatible ρ-termination routes. The ρ factor-dependent termination under riboswitch control or not proceeds via three different but compatible routes. The riboswitch gating by a ligand (green) most affects the stand-by decomposing termination (bottom row) among the three routes. Comparatively, the gating effects on the two catch-up routes (upper rows) are ancillary so simplified here to be absent. It is not known whether and how long ρ remains on the terminated transcript RNA. Moreover, the three routes operate on their distinct timescales, which is more evident here in Supplementary Figure S6 than our previous study (29). The stand-by ρ binds RNAP a priori earlier than the catch-up ρ. However, the stand-by ρ’s sole route for decomposing termination runs last as the slowest and is preceded by the catch-up ρ’s major route for decomposing termination in all the five terminators, although the timing error bars overlap in the *rho* terminator. Nevertheless, the catch-up ρ’s minor route for recycling termination comes clearly first as the fastest in all the terminators. The decomposing outcome is prevailing after ρ-dependent termination and would allow for 3D reinitiation by the reassociated RNAP (29). The recycling outcome renders the DNA-bound RNAP to diffuse on the same template molecule and lead to 1D reinitiation by the recycling RNAP just like after most intrinsic terminations, where the hairpin recycling route is much more frequent than the hairpin decomposing route (37,54).

These findings depicted in Figure 7 are contrary to previous conjectures. First, following the two catch-up routes, ρ first binds an RNA *rut* site and catches up with the paused RNAP, so the pause has to be long enough for the catching-up and has been deemed sensitive to the pause duration. However, the catch-up decomposing ρTE turns out to be little affected by pause extension or reduction in a range of 1.6 to 4.2 min. The *rut* sites are mostly only 10–20 nt away from the 3' end of RNA and rarely farther than 100 nt (49–52). As ρ translocates on RNA at a speed of 56 ± 3 nt/s (53), ρ would take only a few seconds to search a *rut* site and move along RNA by the distance but not much longer. Conclusively, a pause is imperative but does not need to be long for efficient termination via the catch-up routes, and the long pauses observed with many ρ-dependent terminators may have a role for something else.

Second, following the stand-by route, which is generally less frequent than the two catch-up routes combined (29), ρ pre-binds RNAP and stands by for an RNA *rut* site, so the stand-by ρ has been presumed ready for execution and not to need long pauses. However, pause shrinkages to a minute or so hamper the stand-by route. How so? We hypothesize that the RNAP-prebound ρ is inactive or only partially active and takes time to get fully activated. This hypothesis could be consistent with that the RNAP-bound ρ requires conformational changes to capture a *rut* site (24,46). In the *E. coli* ρ hexamer ring, one protomer designated ρ_6_ is bound to another protomer ρ_5_ and NusA factor, but the *rut-*site binding is stabilized only after the ρ_6_ moves by ∼4.5 nm towards another protomer ρ_1_ and RNAP β’ subunit, shifting the ring opening from ρ_6_·ρ_1_ to ρ_5_·ρ_6_ junction. Furthermore, a series of additional conformation changes occur in the ρ·RNAP pre-terminational complex (24).

What can be an advantage of the slow activation for *rut* recognition in the stand-by termination route? Riboswitch RNA folding can be regulated thermodynamically or kinetically. In the kinetic controls, ligand recognition occurs fast as a co-transcriptional process before a thermodynamic equilibrium is reached. In the thermodynamic controls, by contrast, ligand recognition occurs after an equilibrium is established. Looking forward to obtaining more experimental proofs, we postulate that the *mgtA* and *ribB* riboswitch terminators are thermodynamically regulated primarily by utilizing the stand-by ρ’s slow *rut* recognition and long pauses.

Initially in this study, we used a minimal transcription system comprising only linear DNA template, RNAP holoenzyme and the general termination factor ρ. In order to emulate the intracellular environment as much as practically possible, we performed the three supplementary sets of experiments each with addition of NusA/G, competitor RNAs or a crowding agent. We found that the main conclusions of this study concerning the pause-termination correlations remain valid even with the addendum. However, we would not exclude the possibility that other factors such as DNA supercoiling could have a different effect.

In this study, we have shown that the three compatible routes of ρ-dependent termination are not only kinetically different but also play distinct regulatory roles. Including the ρ-free decomposing background termination, it is the four termination routes that operate compatibly together in any ρ-dependent terminator. We and others have previously shown that two termination routes are compatible in bacterial intrinsic terminators, and that the hairpin recycling route is much more frequent than the hairpin decomposing route (37,54). Multiple models have been proposed for each of the eukaryotic RNAP I, II and III terminations (55–57) so it would be interesting to see if they are compatible in a terminator as well. If so, it could be generalized in all types of termination that multiple routes together achieve maximum possible efficiency of termination operating on diverse timescales under separate regulations.

In summary, the three ρ-dependent termination routes dissimilarly depend on transcriptional pauses. The pause duration-termination efficiency correlation is invariably and highly positive only for the stand-by decomposing termination among the three routes, regardless whether NusA/G factors, competitor RNAs or a macromolecular crowder is added or not. Moreover, the two ρ-dependent terminator-associated riboswitches largely regulate the stand-by ρ’s slow termination. These results could provide unprecedented insights about the roles of transcriptional pauses in bacterial ρ-dependent terminations and possibly the general transcription termination mechanism.

## DATA AVAILABILITY

All data are available from the corresponding authors upon reasonable request.

## Supplementary Material

gkad051_Supplemental_File
