# Supplementary material for: Transcriptional pause extension benefits the stand-by rather than catch-up Rho-dependent termination
Source: Nucleic Acids Res. 2023 Feb 10;51(6):2778–89. doi: 10.1093/nar/gkad051 (PMC10085680; doi:10.1093/nar/gkad051)
Supplement: gkad051_Supplemental_File [file gkad051_supplemental_file.docx]

**Supplementary Data**

**Transcriptional pause extension benefits the stand-by rather than catch-up Rho-dependent termination**

Eunho Song^1^, Seungha Hwang^2^, Palinda Ruvan Munasingha^3^, Yeon-Soo Seo^3^,

Jin Young Kang^2,^*, Changwon Kang^3,4,^* and Sungchul Hohng^1,^*

^1^ Department of Physics and Astronomy, and Institute of Applied Physics, Seoul National University, Seoul 08826, Republic of Korea

^2^ Department of Chemistry, Korea Advanced Institute of Science and Technology, Daejeon 34141, Republic of Korea

^3^ Department of Biological Sciences, Korea Advanced Institute of Science and Technology, Daejeon 34141, Republic of Korea

^4^ KAIST Stem Cell Center, Korea Advanced Institute of Science and Technology, Daejeon 34141, Republic of Korea

* To whom correspondence should be addressed. Email: shohng@snu.ac.kr; ckang@kaist.ac.kr; jykang59@kaist.ac.kr

**Contents**

Supplementary Figure S1. Pause and ρ-termination measurements in the time-course bulk assays

Supplementary Figure S2. Invariance of the pause-termination correlations upon variation of NTP incorporation speeds

Supplementary Figure S3. Pause duration measurements of the mgtA terminator mutant templates

Supplementary Figure S4. Background termination levels

Supplementary Figure S5. Dependency of pause and termination efficiency on AU content of the termination-site hybrid

Supplementary Figure S6. Termination and readthrough timings of the three routes from the five terminators

Supplementary Figure S7. Effects of NusA/G factors, competitor RNAs, and a crowder on pauses and termination efficiencies

Supplementary Table S1: Oligonucleotides used for construction of transcription templates

Supplementary Table S2: Rho-dependent termination efficiencies of the three routes

Supplementary Table S3: Timings of the three route terminations and readthrough

**Abbreviation**: PEG, polyethylene glycol; PIFE, protein-induced fluorescence enhancement; TE, termination efficiency; TS, termination site; ρTE, ρ-dependent termination efficiency.

**Supplementary Figure S1.** Pause and ρ-termination measurements in the time-course bulk assays.

**
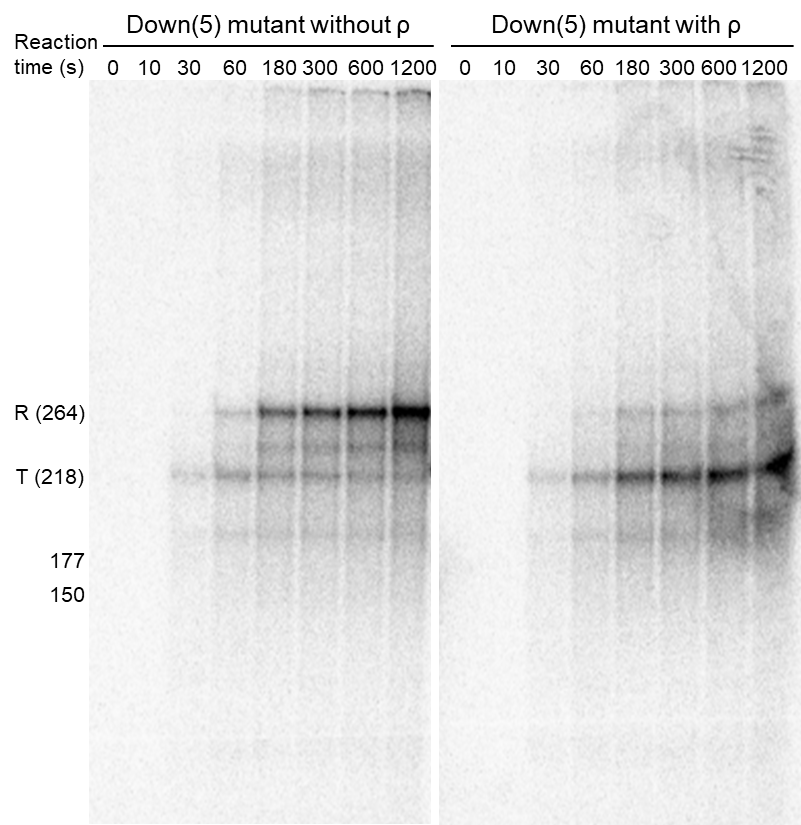
**

**
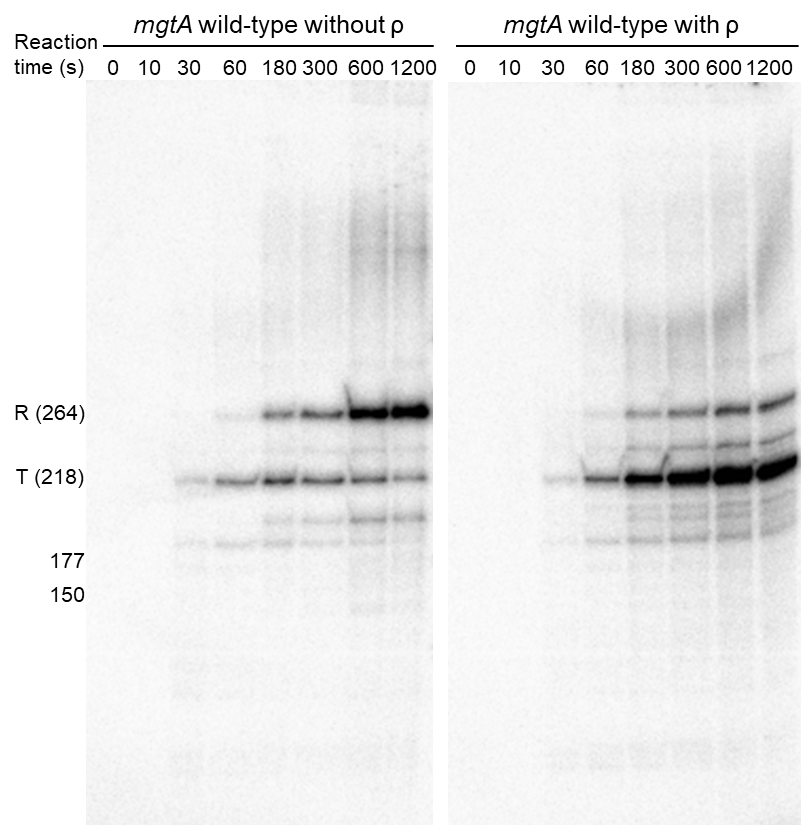
**

**
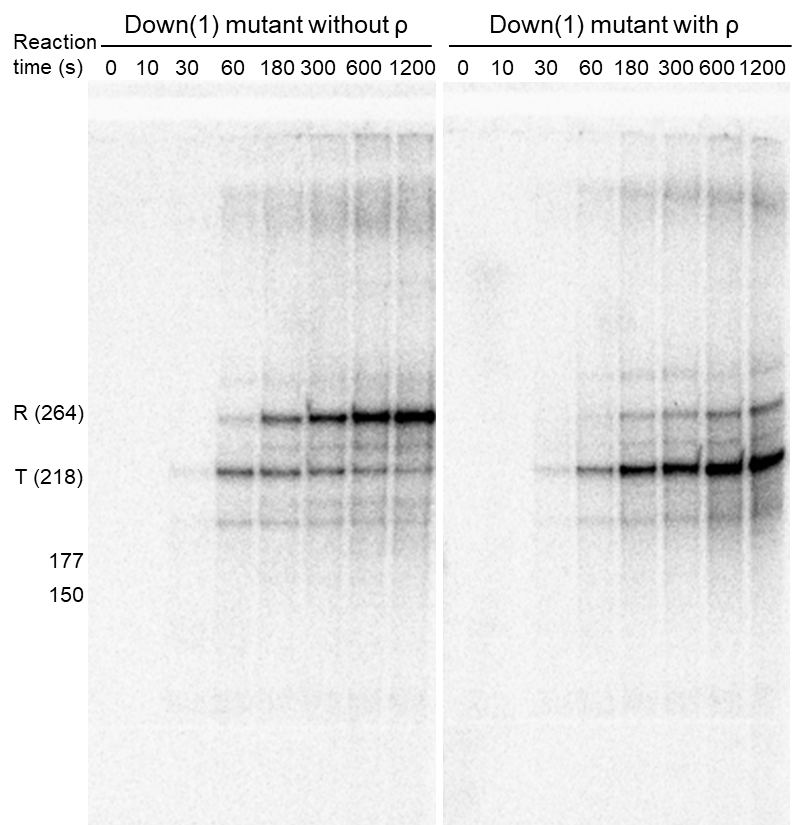
**

Down(5) mutant

Down(1) mutant

AU11 mutant

AU78 mutant

*mgtA* wild-type

**
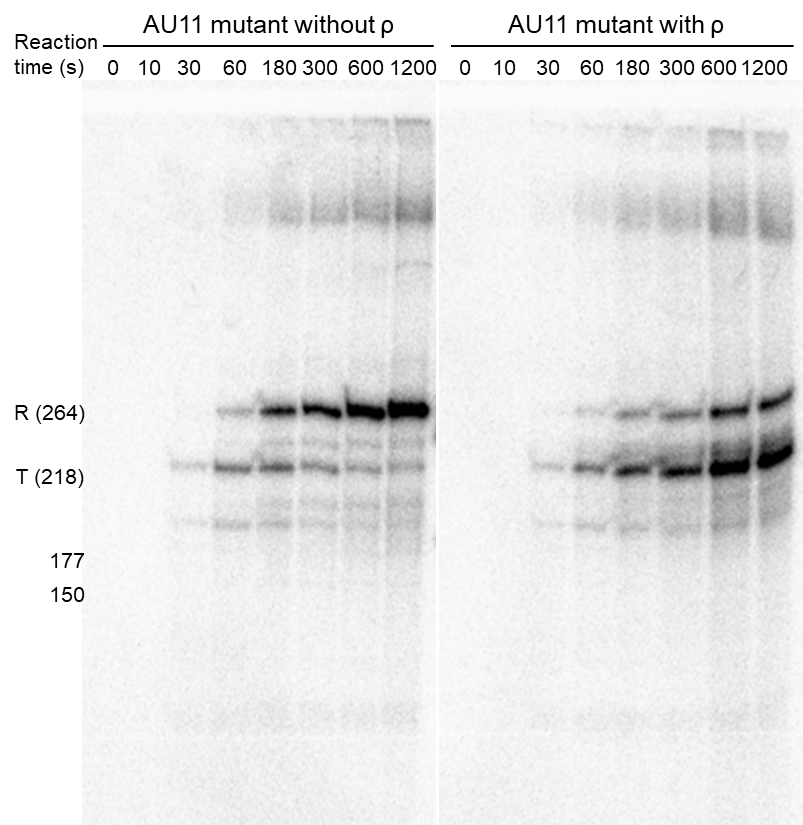
**

**
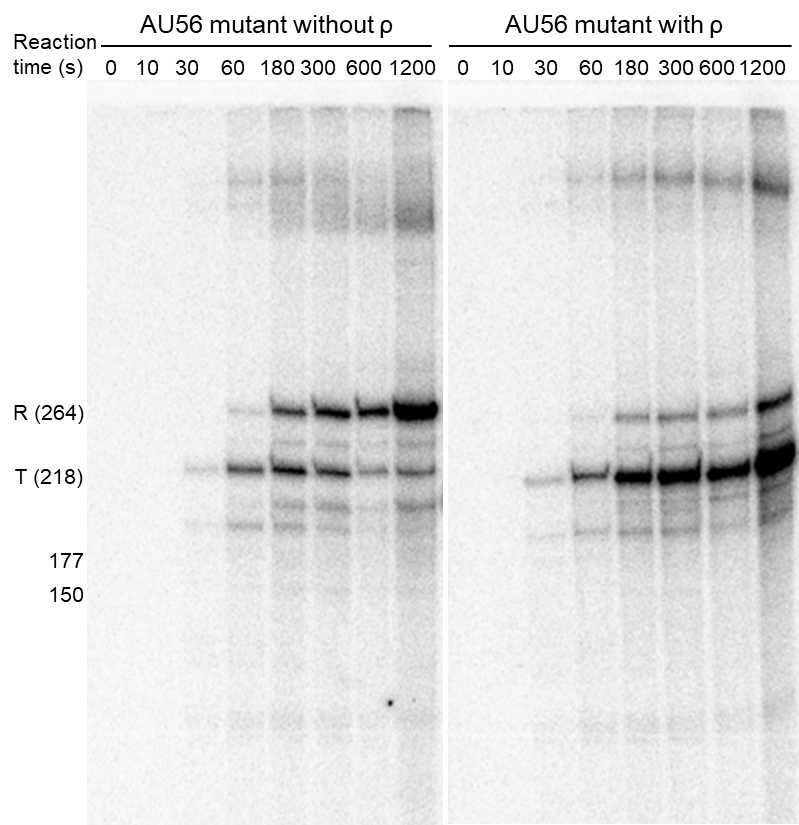
**

**
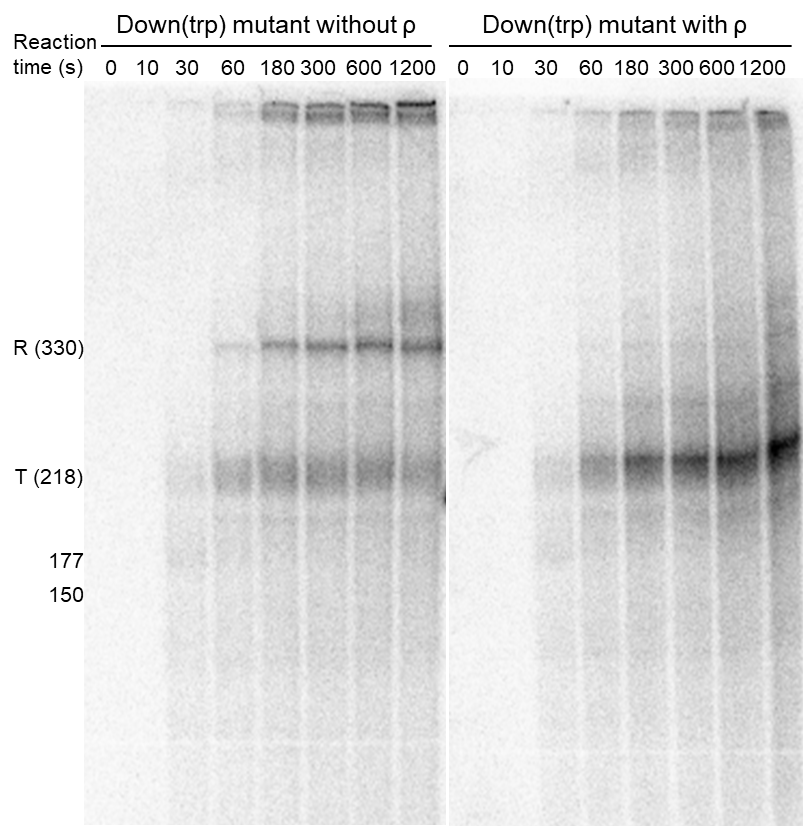
**

AU56 mutant

Down(trp) mutant

**
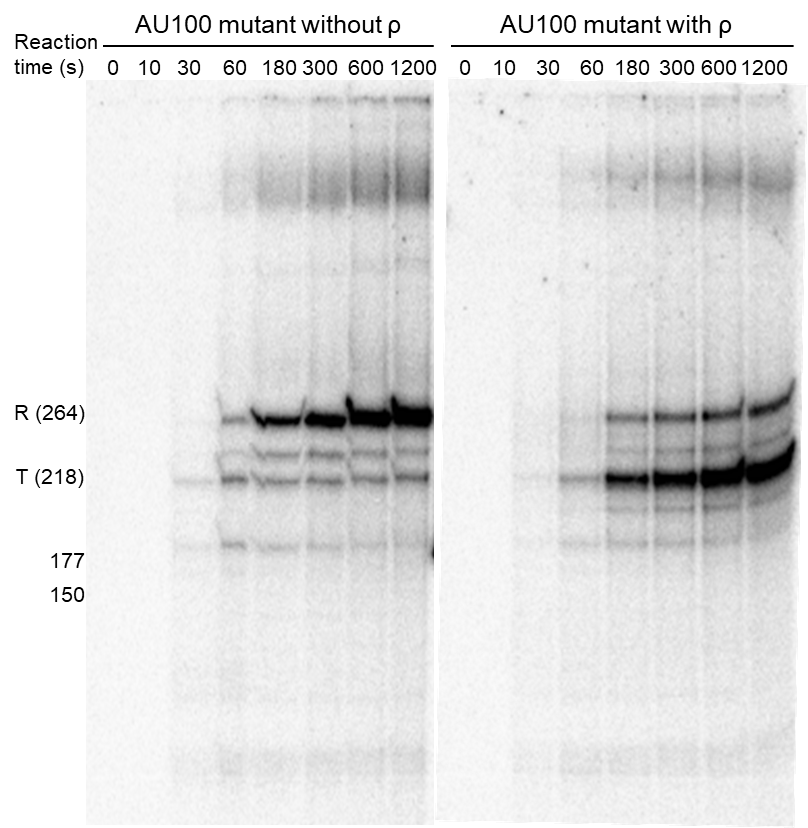

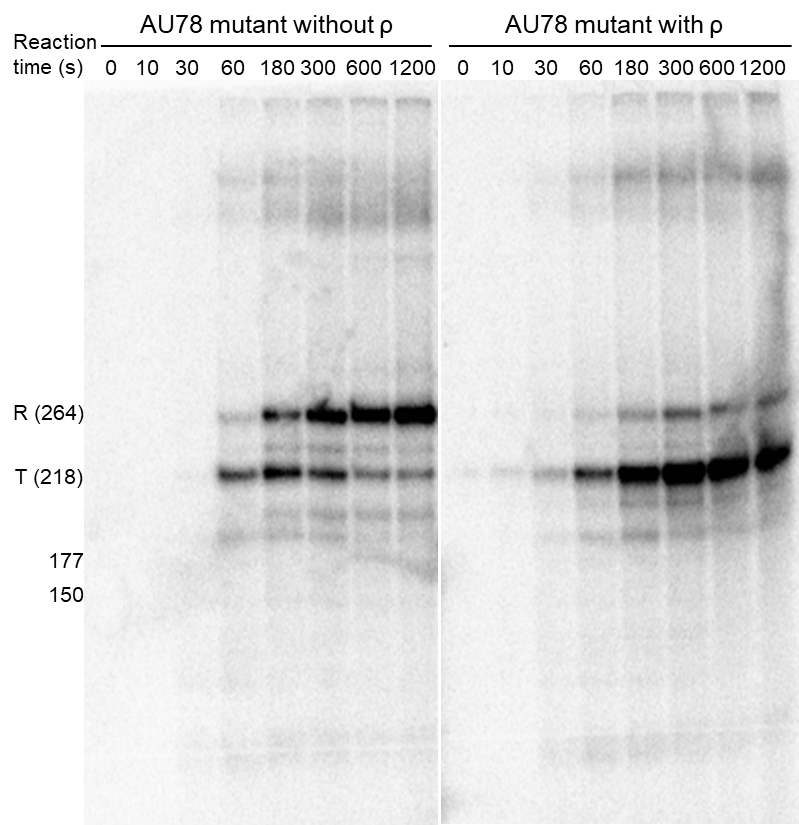

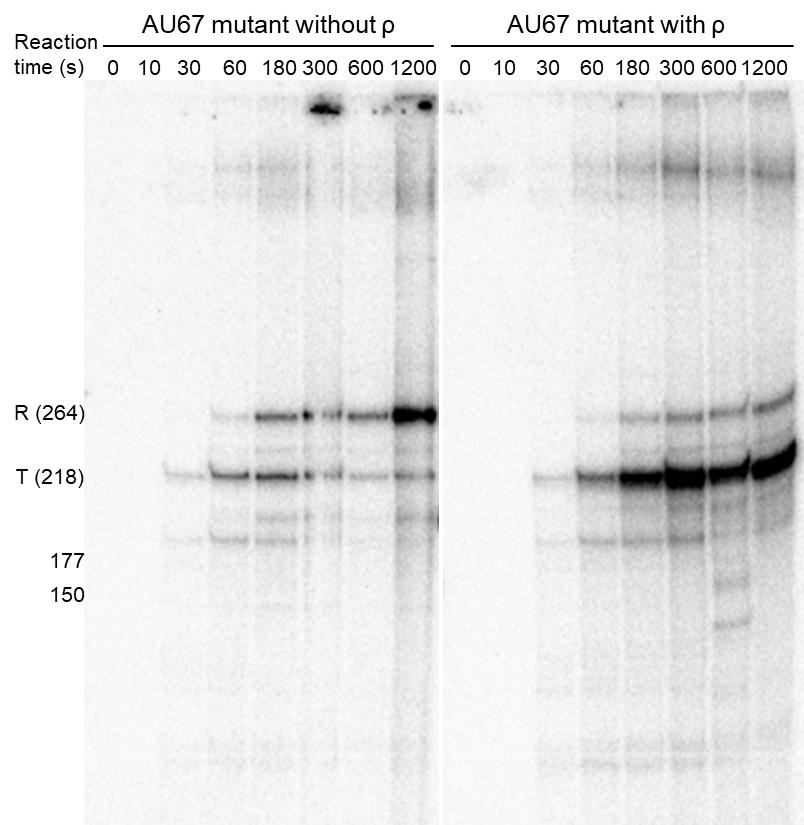
**

AU100 mutant

AU67 mutant

Aliquots were taken at eight timepoints of 0 to 20 min from each bulk transcription reaction of the *mgtA* terminator template or its mutant without or with ρ. The runoff (R) and terminated (T) transcript bands are marked with their nucleotide numbers in parenthesis. The top part of each gel picture shows sample wells and the bottom part devoid of even a faint band is trimmed. In all the experiments without ρ, evident is a strong pause at the T site where termination occurs with ρ. This terminational pause peaks at the 1- or 3-min point in any template. No other pauses are observed except for a very weak one shown just above the 177-nt size standard in some templates. Transcription reactions were each carried out using 30 nM *E. coli* RNAP holoenzyme and 15 nM DNA template with or without 100 nM *E. coli* ρ hexamer in 40 mM Tris-HCl, pH 8.0, 200 μM ATP, 200 μM GTP, 200 μM UTP, 25 μM CTP, 0.75 μCi of [α-^32^P]CTP (3,000 Ci/mmol), 10 mM MgCl_2_, 150 mM KCl, and 2 mM dithiothreitol at 37°C for the indicated reaction time. The reaction products were analyzed by 6% polyacrylamide-7 M urea gel electrophoresis.


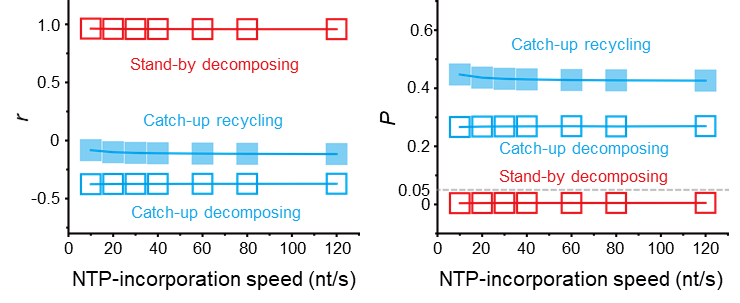
**Supplementary Figure S2.** Invariance of the pause-termination correlations upon variation of NTP incorporation speeds. The Pearson's *r* and *P* values were estimated for the linear correlations between the pause durations and the measured termination efficiencies of the stand-by decomposing (red empty square), catch-up decomposing (blue empty square) and catch-up recycling (blue filled square) termination routes in the five ρ-dependent terminators, while the pause durations were calculated using an indicated NTP incorporation speed without ρ. The two values are separately plotted on the y-axis against the NTP incorporation speeds on the x-axis. A horizontal line in the *P*-value plot indicates a significance threshold α = 0.05. Both values are virtually invariant across a wide range of the incorporation speeds. Conclusively, regardless of what speed is used for calculation of the pause durations, the Pearson's *r* value is close to one and the *P* value is lower than the significance threshold for the pause correlation only with the stand-by decomposing termination.

**Supplementary Figure S3.** Pause duration measurements of the *mgtA* terminator mutant templates.

**
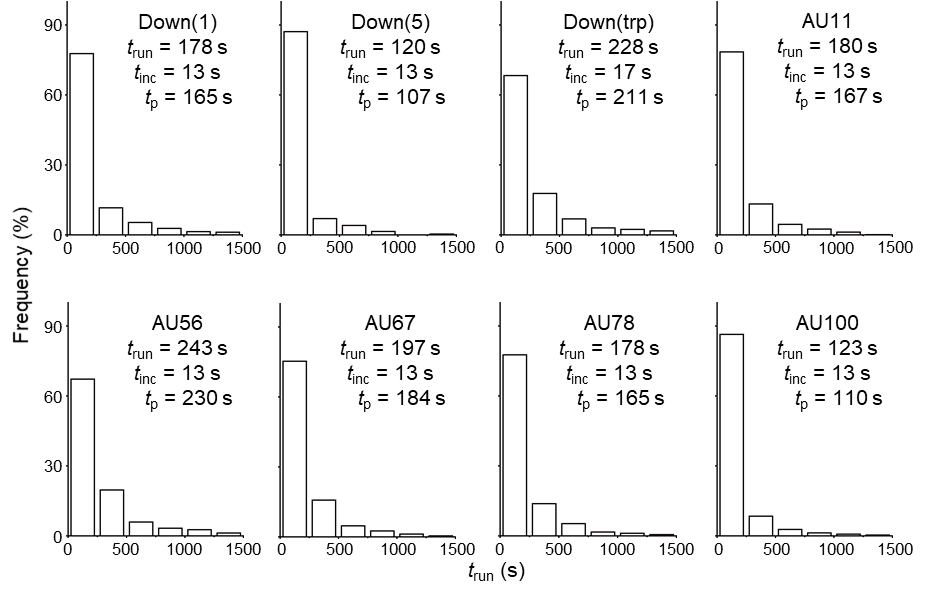
**After the ρ-free elongation timespan (*t*_run_) was experimentally measured in the ρ-free assays and the NTP incorporation timespan (*t*_inc_) was estimated for each *mgtA* terminator mutant template as described in the main text, their pause durations were calculated by *t*_p_ = *t*_run_ – *t*_inc_. The modified basepair sequences of the mutant templates are shown in Figure 4A.

**Supplementary Figure S4.** Background termination levels.


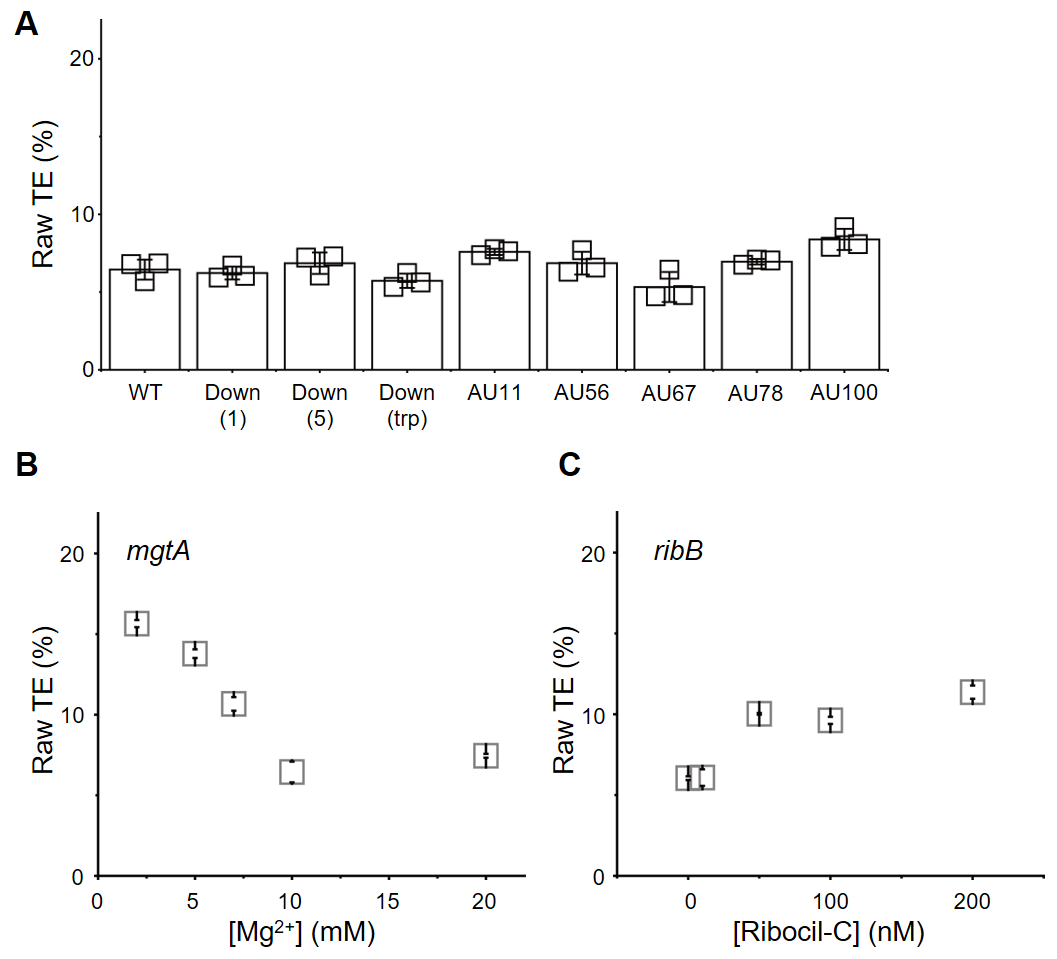
The background termination efficiencies (TEs) were measured without ρ in the ρ-free assays. (**A**) The raw background TEs of the *mgtA* terminator template and its eight mutants. The altered sequences of the mutants are shown in Figure 4A. (**B**) The raw background TEs of the *mgtA* terminator template at varying concentrations of Mg^2+^. (**C**) The raw background TEs of the *ribB* terminator template at varying concentrations of ribocil-C. Error bar represents standard deviation of three independent datasets.

**Supplementary Figure S5.** Dependency of pause and termination efficiency of the catch-up recycling termination on AU content of the termination-site hybrid.


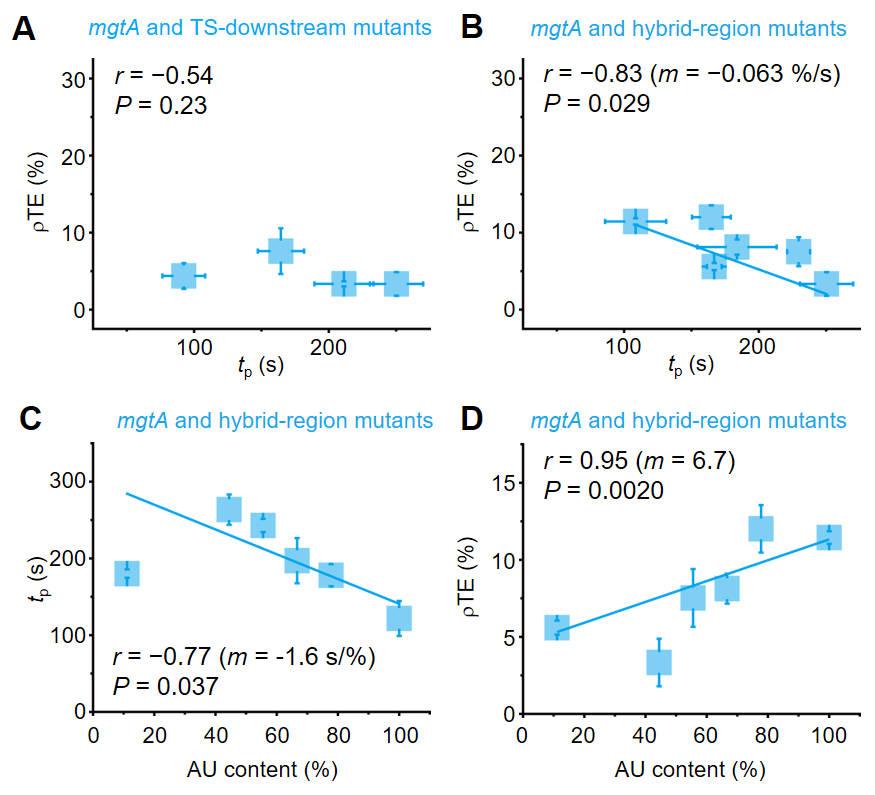
The eight mutants of the *mgtA* terminator template were divided into two groups. The three mutants modified in the termination site (TS)-downstream region were separated from the five mutants varied in the TS-upstream region forming an RNA·DNA hybrid at the major TS. (**A**) Negligible correlation between the ρ-dependent termination efficiency (ρTE) and the pause duration (*t*_p_) in the TS-downstream mutants and the wild-type. (**B**) Correlation between ρTE and *t*_p_ in the hybrid-region mutants and the wild-type. (**C** and **D**) Correlation of *t*_p_ (C) or ρTE (D) with the AU content of the hybrid at TS in the hybrid-region mutants and the wild-type. The Pearson's *r*, *P*-value, and the fitting line slope (*m*) are shown. Error bar represents standard deviation of three independent datasets. The numbers of analyzed molecules are in Supplementary Table S2.

**
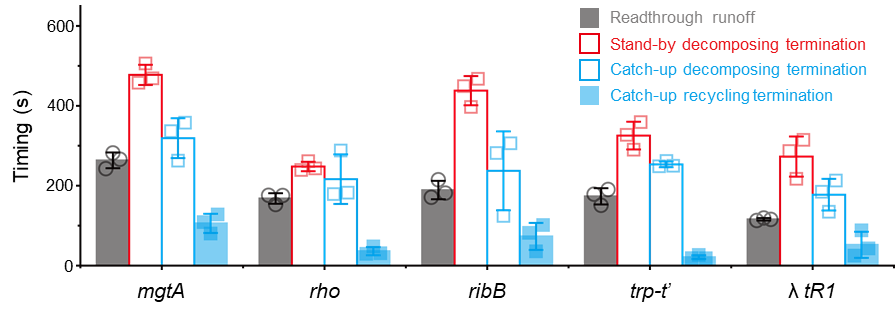
Supplementary Figure S6.** Termination and readthrough timings of the three routes from the five terminators.

Transcription termination was timed as the delay from diminishing of the Cy3's protein-induced fluorescence enhancement (PIFE) to disappearance of the Cy3 signal, and the readthrough runoff was timed as the interval between the Cy3 PIFE diminishing and the Cy5 PIFE appearance in the stand-by and catch-up ρ assays that respectively analyzed 2.9- and 3.6-fold more EC spots (*n* = 5,208 and 6,535, respectively) than our previous paper in 2022 by Song *et al*. in *Nat. Commun.* 13, 1663 (*n* = 1,784 and 1,834, respectively). It is more evident in this study than in the previous study that the three ρ-dependent termination routes operate on their distinct timescales. Among the three routes, the fastest is definitely the catch-up recycling termination (filled blue bars), which takes place much earlier than the readthrough runoff (filled gray bars) in all the five terminators. The second fastest route is the catch-up decomposing termination (empty blue bars), which occurs later than the readthrough runoff, although their timing error bars overlap in some terminators. The slowest route is the stand-by decomposing termination (empty red bars), which completes itself much later than the readthrough runoff. The temporal distinction is clear among the three termination routes in all the terminators, except for that the timing error bars overlap between the two catch-up termination routes of the *rho* terminator. Error bar represents standard deviation of three independent datasets. The numbers of analyzed molecules are in Supplementary Table S2.


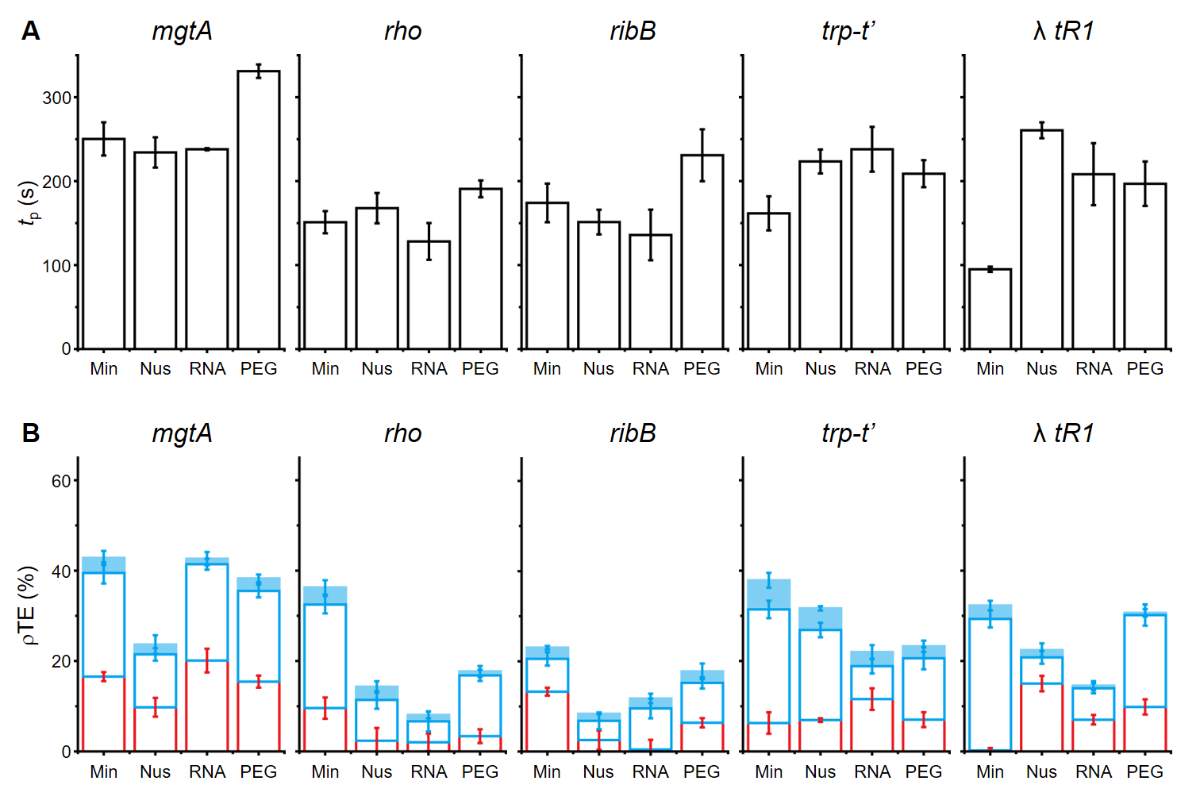
**Supplementary Figure S7.** Effects of NusA/G factors, competitor RNAs, and a crowder on pauses and termination efficiencies.

(**A**) The pause duration *t*_p_ values of the five terminator templates in the minimal system (Min) or with addition of NusA/G factors (Nus), competitor RNAs (RNA), or PEG-8000 (PEG). They were measured in the ρ-free assays as described in Figure 3A. (**B**) The ρ-dependent termination efficiencies (ρTEs) of the recycling (solid) and decomposing (open) termination routes in the minimal system (Min) or with addition of NusA/G (Nus), competitor RNAs (RNA), or PEG-8000 (PEG). They were measured with each of the five terminator templates in the stand-by (red) and catch-up (cyan) ρ assays. Those of the three termination routes are stacked. Error bar represents standard deviation of three independent datasets.

**Supplementary Table S1.** Oligonucleotides^a^ used for construction of transcription templates^b^.

| Name | Length | Sequence in the 5' to 3' direction |
| --- | --- | --- |
| A | 200 nt | TATCAAAAAGAGTATTGACTTAAAGTCTAACCTATAGGATACTTACAGCCATACCGCCACAAAACTTATGGATTTATGCGTATAATCCGCGGCGCAAATTATTTACTTACCGGAGGCGACATGGACCCTGAACCCACCCCTCTCCCGCGATGGAGAATTTTCCTTTTCCGGTAAGCCTGCCTCTGCTGTCTTACCGGTGT |
| AB | 40 nt | TTATTGTGTCACTGTCTTACACACCGGTAAGACAGCAGAG |
| B for  Down(1) | 114 nt | pGTAAGACAGTGACACAATAACGTCCCTGTTTTTATTTAAACATTGCTCATCGGGCAAGGCTTTGCCGTcCCTGAAGAATTTTCTGCGCCTGACTTCGGCGCGGAGGGATTACCT |
| B for  Down(5) | 114 nt | pGTAAGACAGTGACACAATAACGTCCCTGTTTTTATTTAAACATTGCTCATCGGGCAAGGCTTTGCCGTcggacAAGAATTTTCTGCGCCTGACTTCGGCGCGGAGGGATTACCT |
| B for  Down(trp) | 180 nt | pGTAAGACAGTGACACAATAACGTCCCTGTTTTTATTTAAACATTGCTCATCGGGCAAGGCTTTGCCGTtaattgttaatatatccagaatgttcctcaaaatatattttccctctatcttctcgttgcgcttaatttgactaattctcattagcgactaattttaatgagtgtcgacaca |

^a^ The templates containing the Down(1), Down(2) and Down(trp) mutants of *mgtA* terminator were each constructed by ligation of the A fragment and a B fragment with the AB splint. They are 314, 314, and 380 bp long, respectively. The mutated sequences are in lower cases and underlined. The ligation products were amplified by polymerase chain reactions using a 5'-biotin-labeled forward primer 5'-TATCAAAAAGAGTATTGACTTAAAGTCTAA and a 5'-Cy5-labeled backward primer, 5'-AGGTAATCCCTCCGC or 5'-TGTGTCGACACTCAT.

^b^ The other templates each containing one of the following terminators and mutants were prepared as previously described in the Supplementary Table 1 of our previous paper in 2022 by Song *et al*. in *Nat*. *Commun*. 13, 1663: *mgtA* terminator template (314 bp), *rho* terminator template (390 bp), *ribB* terminator template (350 bp), *trp t’* terminator template (286 bp), λ *tR1* terminator template (548 bp), *mgtA*'s AU11 mutant template (314 bp), *mgtA*'s AU56 mutant template (314 bp), *mgtA*'s AU67 mutant template (314 bp), *mgtA*'s AU78 mutant template (314 bp), and *mgtA*'s AU100 mutant template (314 bp).

**Supplementary Table S2.** Rho-dependent termination efficiencies of the three routes. The ρ-dependent termination efficiencies (ρTEs) were estimated by subtracting the ρ-independent background efficiency from the raw efficiencies. *n* means the number of molecules analyzed.

| 1. ρTEs of various terminators (Figure 3B) | | | | |
| --- | --- | --- | --- | --- |
| Terminator | Assay | Decomposing  termination (%) | Recycling  termination (%) | *n* in replicated experiments |
| *mgtA* | Stand-by | 16.6 ± 1.0 | 0.2 ± 0.0 | 1,363 = 420 + 468 + 475 |
|  | Catch-up | 22.9 ± 2.3 | 3.3 ± 1.5 | 1,781 = 577 + 599 + 605 |
| *rho* | Stand-by | 9.6 ± 2.4 | 0.5 ± 0.1 | 1,099 = 156 + 230 + 713 |
|  | Catch-up | 22.9 ± 1.9 | 3.8 ± 1.6 | 1,519 = 611 + 402 + 506 |
| *ribB* | Stand-by | 13.3 ± 0.9 | 0.2 ± 0.3 | 1,181 = 494 + 322 + 365 |
|  | Catch-up | 7.3 ± 1.5 | 2.7 ± 0.4 | 364 = 164 + 131 + 87 |
| *trp-t’* | Stand-by | 6.3 ± 2.4 | 0.3 ± 0.3 | 1,100 = 420 + 435 + 245 |
|  | Catch-up | 25.1 ± 1.9 | 6.5 ± 1.7 | 792 = 280 + 314 + 198 |
| λ *tR1* | Stand-by | 0.2 ± 0.6 | 0.0 ± 0.0 | 422 = 253 + 65 + 104 |
|  | Catch-up | 29.1 ± 1.9 | 3.0 ± 1.0 | 925 = 337 + 315 + 273 |
|  | | | | |
| 2. ρTEs of various mutants of the *mgtA* terminator template (Figure 4C and Supplementary Figure S5) | | | | |
| Mutants | Assay | Decomposing  termination (%) | Recycling  termination (%) | *n* in replicated experiments |
| Down(1) | Stand-by | 16.6 ± 1.0 | 0.2 ± 0.0 | 1,545 = 500 + 561 + 484 |
|  | Catch-up | 25.1 ± 0.9 | 7.6 ± 3.0 | 1,461 = 273 + 540 + 648 |
| Down(5) | Stand-by | 9.9 ± 1.8 | 0.0 ± 0.3 | 476 = 101 + 154 + 221 |
|  | Catch-up | 23.7 ± 2.4 | 4.4 ± 1.7 | 1,736 = 567 + 568 + 601 |
| Down(trp) | Stand-by | 8.2 ± 1.7 | 0.0 ± 0.0 | 604 = 278 + 165 + 161 |
|  | Catch-up | 14.4 ± 2.1 | 3.4 ± 0.3 | 1,044 = 382 + 348 + 314 |
| AU11 | Stand-by | 10.9 ± 2.0 | 0.1 ± 0.1 | 1,294 = 592 + 344 + 358 |
|  | Catch-up | 23.7 ± 1.9 | 5.6 ± 0.5 | 1,324 = 488 + 411 + 425 |
| AU56 | Stand-by | 8.3 ± 2.2 | 0.1 ± 0.1 | 1,366 = 491 + 392 + 483 |
|  | Catch-up | 19.9 ± 1.9 | 7.5 ± 1.9 | 1,465 = 493 + 547 + 425 |
| AU67 | Stand-by | 12.9 ± 1.8 | 0.1 ± 0.2 | 1,244 = 413 + 361 + 470 |
|  | Catch-up | 14.9 ± 2.7 | 8.1 ± 1.0 | 1,568 = 570 + 483 + 515 |
| AU78 | Stand-by | 13.4 ± 0.8 | 0.0 ± 0.0 | 536 = 246 + 137 + 153 |
|  | Catch-up | 27.4 ± 1.0 | 12.0 ± 1.5 | 1,274 = 408 + 496 + 370 |
| AU100 | Stand-by | 1.5 ± 1.3 | 0.0 ± 0.0 | 571 = 251 + 180 + 140 |
|  | Catch-up | 20.6 ± 2.2 | 11.4 ± 0.4 | 1,057 = 291 + 274 + 492 |
|  | | | | |
| 3. ρTEs of *mgtA* at varying Mg^2+^ concentrations (Figure 5A) | | | | |
| [Mg^2+^] | Assay | Decomposing  termination (%) | Recycling  termination (%) | *n* in replicated experiments |
| 2 mM | Stand-by | 4.6 ± 0.9 | -0.3 ± 0.1 | 495 = 194 + 219 + 82 |
|  | Catch-up | 18.3 ± 1.4 | 1.7 ± 1.8 | 498 = 170 + 169 + 159 |
| 5 mM | Stand-by | 3.7 ± 0.4 | 0.0 ± 0.0 | 456 = 163 + 195 + 98 |
|  | Catch-up | 17.6 ± 1.5 | 5.2 ± 2.0 | 1,405 = 832 + 267 + 306 |
| 7 mM | Stand-by | 10.1 ± 0.6 | 0.0 ± 0.0 | 261 = 123 + 66 + 72 |
|  | Catch-up | 19.5 ± 0.5 | 6.4 ± 1.3 | 871 = 278 + 278 + 315 |
| 10 mM | Stand-by | 16.6 ± 1.0 | 0.2 ± 0.0 | 1,363 = 420 + 468 + 475 |
|  | Catch-up | 22.9 ± 2.3 | 3.3 ± 1.5 | 1,781 = 577 + 599 + 605 |
| 20 mM | Stand-by | 18.9 ± 0.9 | 0.0 ± 0.0 | 395 = 182 + 142 + 71 |
|  | Catch-up | 23.8 ± 1.0 | 3.5 ± 1.8 | 629 = 259 + 215 + 155 |
|  | | | | |
| 4. ρTEs of *ribB* at varying ribocil-C concentrations (Figure 5C) | | | | |
| [Ribocil-C] | Assay | Decomposing  termination (%) | Recycling  termination (%) | *n* in replicated experiments |
| 0 nM | Stand-by | 5.7 ± 0.5 | 0.0 ± 0.0 | 1,224 = 301 + 501 + 422 |
|  | Catch-up | 9.9 ± 1.1 | 1.8 ± 0.5 | 1,518 = 562 + 419 + 537 |
| 10 nM | Stand-by | 7.0 ± 0.6 | 0.0 ± 0.0 | 793 = 254 + 153 + 386 |
|  | Catch-up | 10.0 ± 0.9 | 1.8 ± 0.4 | 516 = 267 + 97 + 152 |
| 50 nM | Stand-by | 7.5 ± 0.2 | 0.2 ± 0.4 | 314 = 96 + 143 + 75 |
|  | Catch-up | 8.0 ± 1.1 | 1.1 ± 0.4 | 805 = 412 + 149 + 244 |
| 100 nM | Stand-by | 14.2 ± 1.1 | 0.5 ± 0.3 | 563 = 124 + 160 + 279 |
|  | Catch-up | 7.4 ± 0.3 | 1.1 ± 0.4 | 956 = 238 + 254 + 464 |
| 200 nM | Stand-by | 13.3 ± 0.9 | 0.2 ± 0.3 | 1,181 = 494 + 322 + 365 |
|  | Catch-up | 7.3 ± 1.5 | 2.5 ± 0.4 | 364 = 146 + 131 + 87 |
|  | | | | |

| 5. ρTEs of various terminators with *E. coli* NusA/G (Figure 6A) | | | | |
| --- | --- | --- | --- | --- |
| Terminator | Assay | Decomposing  termination (%) | Recycling  termination (%) | *n* in replicated experiments |
| *mgtA* | Stand-by | 9.8 ± 2.1 | 0.0 ± 0.0 | 553 = 107 + 274 + 172 |
|  | Catch-up | 11.6 ± 1.4 | 2.2 ± 2.0 | 473 = 272 + 88 + 113 |
| *rho* | Stand-by | 2.4 ± 2.8 | 0.0 ± 0.0 | 564 = 226 + 154 + 184 |
|  | Catch-up | 9.0 ± 2.0 | 2.9 ± 1.2 | 492 = 163 + 134 + 195 |
| *ribB* | Stand-by | 2.5 ± 2.1 | 0.5 ± 0.5 | 579 = 228 + 169 + 182 |
|  | Catch-up | 4.3 ± 1.9 | 1.6 ± 0.1 | 387 = 122 + 138 + 127 |
| *trp-t’* | Stand-by | 6.9 ± 0.4 | 0.4 ± 0.6 | 537 = 89 + 219 + 229 |
|  | Catch-up | 19.9 ± 1.6 | 4.8 ± 0.5 | 489 = 193 + 166 + 130 |
| λ *tR1* | Stand-by | 15.0 ± 1.7 | 0.0 ± 0.0 | 321 = 134 + 56 + 131 |
|  | Catch-up | 5.8 ± 1.4 | 1.6 ± 1.5 | 274 = 102 + 104 + 68 |
|  | | | | |

| 6. ρTEs of various terminators with *E. coli* total RNA (Figure 6B) | | | | |
| --- | --- | --- | --- | --- |
| Terminator | Assay | Decomposing  termination (%) | Recycling  termination (%) | *n* in replicated experiments |
| *mgtA* | Stand-by | 20.1 ± 2.6 | 0.0 ± 0.0 | 352 = 92 + 136 + 124 |
|  | Catch-up | 21.3 ± 1.2 | 1.2 ± 1.5 | 350 = 105 + 119 + 126 |
| *rho* | Stand-by | 2.0 ± 1.9 | 0.5 ± 1.8 | 255 = 124 + 134 + 101 |
|  | Catch-up | 4.6 ± 2.2 | 1.4 ± 0.7 | 473 = 172 + 155 + 146 |
| *ribB* | Stand-by | 0.5 ± 2.1 | 0.0 ± 0.0 | 409 = 176 + 97 + 136 |
|  | Catch-up | 9.1 ± 2.2 | 2.2 ± 1.1 | 582 = 206 + 215 + 161 |
| *trp-t’* | Stand-by | 11.6 ± 2.4 | 0.7 ± 0.1 | 433 = 150 + 162 + 121 |
|  | Catch-up | 7.3 ± 1.6 | 3.1 ± 1.5 | 500 = 143 + 175 + 182 |
| λ *tR1* | Stand-by | 7.0 ± 1.1 | 0.0 ± 0.0 | 365 = 178 + 98 + 89 |
|  | Catch-up | 7.0 ± 1.1 | 0.6 ± 1.0 | 326 = 107 + 115 + 104 |
|  | | | | |

| 7. ρTEs of various terminators with polyethylene glycol 8000 (Figure 6C) | | | | |
| --- | --- | --- | --- | --- |
| Terminator | Assay | Decomposing  termination (%) | Recycling  termination (%) | *n* in replicated experiments |
| *mgtA* | Stand-by | 15.5 ± 1.3 | 0.0 ± 0.0 | 444 = 126 + 117 + 201 |
|  | Catch-up | 20.1 ± 1.4 | 2.8 ± 0.9 | 374 = 154 + 111 + 109 |
| *rho* | Stand-by | 3.4 ± 1.5 | 0.0 ± 0.8 | 338 = 108 + 151 + 79 |
|  | Catch-up | 13.5 ± 1.2 | 0.9 ± 1.2 | 264 = 94 + 101 + 69 |
| *ribB* | Stand-by | 6.4 ± 1.0 | -0.2 ± 0.4 | 398 = 191 + 109 + 98 |
|  | Catch-up | 8.8 ± 1.2 | 2.6 ± 1.7 | 469 = 151 + 147 + 171 |
| *trp-t’* | Stand-by | 7.0 ± 1.6 | 0.3 ± 0.7 | 491 = 161 + 187 + 143 |
|  | Catch-up | 13.6 ± 2.5 | 2.7 ± 1.2 | 463 = 189 + 145 + 129 |
| λ *tR1* | Stand-by | 9.8 ± 1.7 | 0.0 ± 0.0 | 280 = 90 + 82 + 108 |
|  | Catch-up | 20.3 ± 2.4 | 0.5 ± 0.8 | 244 = 70 + 108 + 66 |
|  | | | | |

**Supplementary Table S3.** Timings of the three route terminations and readthrough.

| 1. Timings of various terminators (Supplementary Figure S6) | | | | |
| --- | --- | --- | --- | --- |
| Terminator | Assay | Pathway | Timing (s) | *n* in replicated experiments |
| *mgtA* | Rho-free | Runoff | 264 ± 20 | 387 = 96 + 109 + 182 |
|  | Stand-by | Decomposing | 477 ± 25 | 314 = 93 + 109 + 112 |
|  | Catch-up | Decomposing | 319 ± 50 | 523 = 172 + 188 + 163 |
|  | Catch-up | Recycling | 106 ± 24 | 59 = 29 + 12 + 18 |
| *rho* | Rho-free | Runoff | 168 ± 13 | 116 = 17 + 77 + 22 |
|  | Stand-by | Decomposing | 248 ± 12 | 157 = 27 + 38 + 92 |
|  | Catch-up | Decomposing | 217 ± 62 | 442 = 190 + 113 + 139 |
|  | Catch-up | Recycling | 36 ± 10 | 55 = 12 + 18 + 25 |
| *ribB* | Rho-free | Runoff | 189 ± 23 | 159 = 53 + 51 + 55 |
|  | Stand-by | Decomposing | 438 ± 37 | 296 = 128 + 79 + 89 |
|  | Catch-up | Decomposing | 237 ± 99 | 69 = 26 + 27 + 16 |
|  | Catch-up | Recycling | 73 ± 34 | 10 = 4 + 4 + 2 |
| *trp-t’* | Rho-free | Runoff | 173 ± 20 | 163 = 70 + 56 + 37 |
|  | Stand-by | Decomposing | 325 ± 35 | 137 = 42 + 60 + 35 |
|  | Catch-up | Decomposing | 253 ± 7 | 251 = 94 + 98 + 59 |
|  | Catch-up | Recycling | 21 ± 5 | 53 = 23 + 23 + 9 |
| λ *tR1* | Rho-free | Runoff | 116 ± 3 | 27 = 8 + 15 + 4 |
|  | Stand-by | Decomposing | 273 ± 50 | 33 = 20 + 5 + 8 |
|  | Catch-up | Decomposing | 178 ± 40 | 339 = 127 + 109 + 103 |
|  | Catch-up | Recycling | 52 ± 33 | 28 = 14 + 7 + 7 |
|  | | | | |
| 2. Timings of various mutants of the *mgtA* terminator template (Supplementary Figure S3) | | | | |
| Terminator | Assay | Pathway | Timing (s) | *n* in replicated experiments |
| Down(1) | Rho-free | Runoff | 178 ± 17 | 431 = 140 + 111 + 180 |
| Down(5) | Rho-free | Runoff | 120 ± 16 | 272 = 93 + 90 + 89 |
| Down(trp) | Rho-free | Runoff | 227 ± 22 | 467 = 142 + 84 + 241 |
| AU11 | Rho-free | Runoff | 180 ± 5 | 1,086 = 666 + 206 + 214 |
| AU56 | Rho-free | Runoff | 242 ± 9 | 488 = 199 + 96 + 193 |
| AU67 | Rho-free | Runoff | 197 ± 29 | 907 = 247 + 297 + 363 |
| AU78 | Rho-free | Runoff | 178 ± 14 | 362 = 105 + 119 + 138 |
| AU100 | Rho-free | Runoff | 123 ± 22 | 1,088 = 525 + 256 + 307 |

| 3. Timings of various terminators with *E. coli* NusA/G (Supplementary Figure S7A) | | | | |
| --- | --- | --- | --- | --- |
| Terminator | Assay | Pathway | Timing (s) | *n* in replicated experiments |
| *mgtA* | Rho-free | Runoff | 247 ± 18 | 490 = 170 + 189 + 131 |
| *rho* | Rho-free | Runoff | 185 ± 18 | 637 = 172 + 239 + 226 |
| *ribB* | Rho-free | Runoff | 166 ± 15 | 469 = 149 + 160 + 160 |
| *trp-t’* | Rho-free | Runoff | 235 ± 14 | 499 = 121 + 174 + 204 |
| λ *tR1* | Rho-free | Runoff | 282 ± 10 | 896 = 420 + 173 + 303 |

| 4. Timings of various terminators with *E. coli* total RNA (Supplementary Figure S7B) | | | | |
| --- | --- | --- | --- | --- |
| Terminator | Assay | Pathway | Timing (s) | *n* in replicated experiments |
| *mgtA* | Rho-free | Runoff | 251 ± 1 | 955 = 562 + 286 + 107 |
| *rho* | Rho-free | Runoff | 145 ± 22 | 396 = 149 + 109 + 138 |
| *ribB* | Rho-free | Runoff | 151 ± 30 | 591 = 212 + 188 + 191 |
| *trp-t’* | Rho-free | Runoff | 250 ± 27 | 643 = 171 + 237 + 235 |
| λ *tR1* | Rho-free | Runoff | 229 ± 37 | 487 = 229 + 116 + 142 |

| 5. Timings of various terminators with polyethylene glycol 8000 (Supplementary Figure S7C) | | | | |
| --- | --- | --- | --- | --- |
| Terminator | Assay | Pathway | Timing (s) | *n* in replicated experiments |
| *mgtA* | Rho-free | Runoff | 344 ± 8 | 504 = 215 + 152 + 137 |
| *rho* | Rho-free | Runoff | 208 ± 10 | 338 = 140 + 105 + 93 |
| *ribB* | Rho-free | Runoff | 246 ± 31 | 544 = 164 + 237 + 143 |
| *trp-t’* | Rho-free | Runoff | 221 ± 16 | 402 = 163 + 142 + 97 |
| λ *tR1* | Rho-free | Runoff | 218 ± 27 | 330 = 77 + 116 + 137 |
